# Ultrastructural, Cytochemical, and Comparative Genomic Evidence of Peroxisomes in Three Genera of Pathogenic Free-Living Amoebae, Including the First Morphological Data for the Presence of This Organelle in Heteroloboseans

**DOI:** 10.1093/gbe/evaa129

**Published:** 2020-06-30

**Authors:** Arturo González-Robles, Mónica González-Lázaro, Anel Edith Lagunes-Guillén, Maritza Omaña-Molina, Luis Fernando Lares-Jiménez, Fernando Lares-Villa, Adolfo Martínez-Palomo

**Affiliations:** 1 Departamento de Infectómica y Patogénesis Molecular, Centro de Investigación y de Estudios Avanzados del IPN, Ciudad de México, Mexico; 2 Facultad de Estudios Superiores Iztacala, Universidad Nacional Autónoma de México, Tlanepantla, Estado de México, Mexico; 3 Departamento de Ciencias Agronómicas y Veterinarias, Instituto Tecnológico de Sonora, Ciudad Obregón, Sonora, Mexico

**Keywords:** *Acanthamoeba*, *Balamuthia*, *Naegleria*, transmission electron microscopy, diaminobenzidine, peroxin content

## Abstract

Peroxisomes perform various metabolic processes that are primarily related to the elimination of reactive oxygen species and oxidative lipid metabolism. These organelles are present in all major eukaryotic lineages, nevertheless, information regarding the presence of peroxisomes in opportunistic parasitic protozoa is scarce and in many cases it is still unknown whether these organisms have peroxisomes at all. Here, we performed ultrastructural, cytochemical, and bioinformatic studies to investigate the presence of peroxisomes in three genera of free-living amoebae from two different taxonomic groups that are known to cause fatal infections in humans. By transmission electron microscopy, round structures with a granular content limited by a single membrane were observed in *Acanthamoeba castellanii*, *Acanthamoeba griffini*, *Acanthamoeba polyphaga*, *Acanthamoeba royreba*, *Balamuthia mandrillaris* (Amoebozoa), and *Naegleria fowleri* (Heterolobosea). Further confirmation for the presence of peroxisomes was obtained by treating trophozoites in situ with diaminobenzidine and hydrogen peroxide, which showed positive reaction products for the presence of catalase. We then performed comparative genomic analyses to identify predicted peroxin homologues in these organisms. Our results demonstrate that a complete set of peroxins—which are essential for peroxisome biogenesis, proliferation, and protein import—are present in all of these amoebae. Likewise, our in silico analyses allowed us to identify a complete set of peroxins in *Naegleria lovaniensis* and three novel peroxin homologues in *Naegleria gruberi*. Thus, our results indicate that peroxisomes are present in these three genera of free-living amoebae and that they have a similar peroxin complement despite belonging to different evolutionary lineages.

**Significance**

Peroxisomes are single membrane-bound organelles that perform diverse metabolic functions. Since these organelles have been mainly studied in mammals, yeasts, and plants, there is still little information regarding the presence of peroxisomes in other eukaryotic lineages. Our ultrastructural observations revealed the presence of spherical structures limited by a single membrane in free-living amoebae from two different phylogenetic groups, the Amoebozoa and the Heterolobosea. These structureswere selectively stainedwhen cytochemical assayswere performed to detect the presence of catalase, the hallmark enzyme of peroxisomes.We also identified a complete set of peroxins—which are proteins that are present in all peroxisomes regardless of their metabolic content—in all of these Amoebozoans and Heteroloseans, confirming that peroxisomes are present in these free-living amoebae.

## Introduction

Free-living amoebae are widely distributed in nature. These cosmopolitan protozoa have been found in soil (Reyes-Batlle et al. 2014) and aquatic environments such as ponds and swimming pools (Aghajani et al. 2016). Of the many free-living amoeba described to date, three genera from two different taxonomic groups have gained medical importance, as they have been found to be the etiological agents of various human infections. *Naegleria fowleri* (Excavates: Discoba; Heterolobosea; Vahlkampfiidae) (Adl et al. 2019) can produce a highly lethal infection of the central nervous system (CNS) known as primary amoebic meningoencephalitis (Carter 1972). *Balamuthia mandrillaris* (Amoebozoa: Discosea; Centramoebia; Acanthopodida) (Adl et al. 2019) is responsible for the so-called balamuthiasis, a lethal lesion of the CNS that occurs in various animals and can occasionally be observed in humans (Visvesvara et al. 1993). In addition, different species of *Acanthamoeba* (Amoebozoa: Discosea; Centramoebia; Acanthopodida) (Adl et al. 2019) such as *A. castellanii*, *A. polyphaga, A. culbertsoni*, and *A. palestinensis* can produce a subacute or chronic CNS infection known as granulomatous amoebic encephalitis (Martínez 1991; Marciano-Cabral et al. 2000). *Acanthamoeba castellanii* can also produce acanthamoebic keratitis, a sight-threatening infection of the cornea which can occur both in immunocompromised and healthy individuals (Niederkorn et al. 1999; Sharma et al. 2004). Other *Acanthamoeba* species such as *A. royreba* (González-Robles et al. 2013; Omaña-Molina et al. 2016), *A. polyphaga* (Dini et al. 2000; da Rocha-Azevedo and Costa e Silva-Filho 2007), and *A. griffini* (Ledee et al. 1996; González-Robles et al. 2014) have also been isolated from keratitis cases. The leading risk factor for acanthamoebic keratitis is the use of contact lens, as more than 85% of the patients diagnosed with this infection are contact lens users (Cheung et al. 2016; Padhi et al. 2017).

Peroxisomes, also referred to as microbodies, are small organelles that are present in almost all eukaryotic cells. The basic function of peroxisomes is the β-oxidation of fatty acids and the elimination of reactive oxygen species; however, there is a considerable variation in the enzymatic content of these organelles in different organisms. For example, the enzymes of fungal peroxisomes are involved in the synthesis of antibiotics, whereas the peroxisomes of trypanosomatids contain enzymes that perform various glycolytic reactions (Pieuchot and Jedd 2012).

Although the functions and enzymatic content of peroxisomes can vary greatly, all peroxisomes share the same biogenesis route. The different processes involved in the biogenesis of peroxisomes, such as the insertion of proteins into the membrane of the organelle and the import of matrix proteins, are carried out by a group of proteins known as peroxins (Distel et al. 1996). To date, 36 different peroxins have been identified in protists, fungi, plants, and mammals (Galland and Michels 2010; Islinger et al. 2018).

Morphologically, peroxisomes are usually ovoid to spherical, with a size ranging from 0.1 to 1 µm or even 1.5 µm in diameter. These organelles are delimited by a single membrane that encloses a fine fibrogranular matrix which contains numerous enzymes (Smith and Aitchison 2013). The high protein concentration in the peroxisomal matrix can sometimes result in the formation of crystalline inclusions (Sibirny 2016). Biochemically, peroxisomes were first identified by the association of various H_2_O_2_-producing oxidases with catalase, an enzyme that degrades hydrogen peroxide into water and molecular oxygen (De Duve and Baudhuin 1966). The alkaline 3,3′-diaminobenzidine (DAB) reaction for catalase is considered as a specific cytochemical staining for the presence of peroxisomes, and this technique has routinely been used to detect peroxisomes by light and electron microscopy (Schrader and Fahimi 2008).

Data regarding the presence of peroxisomes in free-living amoebae are variable. Müller and MØller (1969) and Childs (1973a) performed biochemical assays that demonstrated the presence of peroxisomes in *Acanthamoeba* and *Hartmanella culbertsoni*, but there is no information regarding the existence of this organelle in *B. mandrillaris.* As for *N. fowleri*, there is no biochemical or morphological evidence for the presence of peroxisomes in this Heterolobosean. However, a couple of studies identified several proteins with peroxisomal targeting signals as well as various putative peroxin homologues (Pex1, 3, 4, 5, 6, 7, 10, 12, and 19) in *Naegleria gruberi*, a nonpathogenic relative of *N. fowleri* (Fritz-Laylin et al. 2010; Opperdoes et al. 2011). Schlüter et al. (2006) proposed that four of these peroxins (Pex3, 10, 12, and 19) could be considered as unequivocal markers for the in silico identification of peroxisomes in eukaryotic cells after observing that they are present in all organisms with peroxisomes and absent in those that lack these organelles. Nonetheless, recent studies in various protozoan lineages have found that at least one of these markers (Pex3) is absent in organisms that are predicted to have peroxisomes (Opperdoes et al. 2016; Ludewig-Klingner et al. 2018; Paight et al. 2019), which suggests that this peroxin may not be an unequivocal indicator for the presence of peroxisomes.

The present study investigated the existence of peroxisomes in *A. castellanii*, *A. griffini*, *A. polyphaga*, *A. royreba*, and *B. mandrillaris* (Amoebozoa) as well as in *N. fowleri* and *Naegleria lovaniensis* (Heterolobosea). Our morphological and cytochemical observations demonstrate that peroxisomes are present in these organisms. These experimental results are further supported by comparative genomic analyses, which allowed us to identify a complete set of predicted peroxins that are necessary for peroxisome biogenesis and maintenance in these Amoebozoans and Heteroloboseans. Additionally, we identified several putative catalase homologues with predicted peroxisomal targeting signals in all of these amoebae, further supporting our cytochemical results. Thus, although they are phylogenetically distant, these free-living amoebae contain peroxisomes and a complete set of peroxins.

## Materials and Methods

### Amoebae

*Naegleria fowleri* and four *Acanthamoeba* species (*A. castellanii*, *A. griffini*, *A. polyphaga* and *A. royreba*) were grown in borosilicate tubes (Pyrex, Mexico) at 37 and 30 °C, respectively, and maintained in axenic culture in 2% Bacto Casitone culture medium (pancreatic digest of casein, Becton Dickinson, Sparks, MD) supplemented with 10% fetal bovine serum (JR Scientific Inc). Trophozoites were harvested during the logarithmic phase of growth (72 h) by chilling the culture tubes in an ice-water bath for 5 min and they were pelleted by centrifugation at 280 × g for 5 min.

Axenic *Balamuthia mandrillaris* trophozoites from the ITSON-1 strain (Lares-Jiménez et al. 2014) were grown in 25 cm^2^ cell culture flasks (TPP, Switzerland) in 4.5 ml of BM-3 culture medium (Schuster and Visvesvara 1996) supplemented with 10% fetal serum. Cultures were incubated at 37 °C in a 5% CO_2_ atmosphere until they reached confluence. Afterwards, the culture medium was carefully removed and the amoebae were washed with phosphate buffered saline pH 7.2 which was previously heated to incubation temperature.

### Cytochemical Staining

To detect catalase activity, we used a modified technique proposed by Beard and Novikoff (1969). Briefly, samples were fixed with 2.5% glutaraldehyde in 0.1 M sodium cacodylate buffer pH 7.2 for 1 h at room temperature. After fixation, the cells were washed twice with 0.1 M cacodylate buffer and incubated for 60 min at 37 °C in an incubation medium containing 0.05 M propanediol buffer pH 10 (Merck-Schuchardt) supplemented with 20 mg/10 ml of 3,3′-diaminobenzidine tetrahydrochloride (Sigma-Aldrich) and 0.2% H_2_O_2_. This solution was filtered and the pH was adjusted to 9 prior to incubation. Mouse liver samples were used as a positive control to detect peroxisome activity. To verify the specificity of the reaction, mouse liver samples were subjected to the same procedure without the addition of 0.2% H_2_O_2_.

### Electron Microscopy

Following incubation, cells were washed twice with 0.1 M cacodylate buffer and postfixed for 1 h with 1% osmium tetroxide in cacodylate buffer. Trophozoites were pelleted by centrifugation, dehydrated, and embedded in polybed resins. Afterwards, samples were polymerized at 60 °C for 24 h. Thin sections (70 nm) were stained with uranyl acetate and lead citrate and examined in a JEOL JEM-1011 transmission electron microscope.

### Comparative Genomic Analyses

To identify putative peroxin proteins in the various Amoebozoans and Heteroloboseans included in our study, the protein sequences of the peroxins Pex1–3, 5–7, 10, 11a–c, 12–14, 16, 19, and 26 from *Homo sapiens* as well as the peroxin protein sequences for Pex1, 3–7, 10–12, 19, and 22 from *N. gruberi* were retrieved from the UniProt database (https://www.uniprot.org/; last accessed July 1, 2020; see supplementary table 1, Supplementary Material online, for accession numbers) (UniProt Consortium 2019) and used as queries for BlastP searches (https://amoebadb.org/amoeba/showQuestion.do?questionFullName=UniversalQuestions.UnifiedBlast; last accessed July 1, 2020; *E-*value cutoff: 1e-04) (Altschul et al. 1990) against the *A. castellanii* and the *N. fowleri* protein databases stored in AmoebaDB, release 46 (https://amoebadb.org/amoeba/; last accessed July 1, 2020) (Aurrecoechea et al. 2011) or for TBlastN searches (https://blast.ncbi.nlm.nih.gov/Blast.cgi?PROGRAM=tblastn&PAGE_TYPE=BlastSearch; TBlastN 2.8.1+; last accessed July 1, 2020; *E-*value cutoff: 1e-04) (Altschul et al. 1997) against the *B. mandrillaris* whole-genome shotgun (WGS) contigs (https://www.ncbi.nlm.nih.gov/nuccore/LEOU00000000.1 and https://www.ncbi.nlm.nih.gov/nuccore/LFUI00000000.1) stored in the NCBI WGS database (https://www.ncbi.nlm.nih.gov/Traces/wgs/?view=wgs; last accessed July 1, 2020) (Sayers et al. 2019). The protein sequences of the *A. castellanii* peroxins identified in this study were then used as queries to perform TBlastN searches against the *A. polyphaga* (https://www.ncbi.nlm.nih.gov/nuccore/CDFK00000000.1 and https://www.ncbi.nlm.nih.gov/nuccore/LQHA00000000.1) and *A. royreba* (https://www.ncbi.nlm.nih.gov/nuccore/CDEZ00000000.1) WGS contigs available in the NCBI WGS database. Similarly, the peroxin protein sequences from *N. fowleri* identified in this study, as well as those from *N. gruberi* and from *Dictyostelium discoideum* (see supplementary table 1, Supplementary Material online, for accession numbers) were used as queries for TBlastN analyses against the *N. lovaniensis* WGS contigs (https://www.ncbi.nlm.nih.gov/nuccore/PYSW00000000.1) deposited in the NCBI WGS database.

**Table 1:** Summary of Predicted Peroxisomal Proteins Identified in Three Genera of Pathogenic Free-Living Amoebae

Protein	Amoebozoa: Discosea, Centramoebia, Acanthopodida				Excavates: Discoba, Heterolobosea, Vahlkampfiidae		
	*Acanthamoeba castellanii*	*Acanthamoeba polyphaga*	*Acanthamoeba royreba*	*Balamuthia mandrillaris*	*Naegleria gruberi*	*Naegleria fowleri*	*Naegleria lovaniensis*
**PEX1**	**+**	**+**	**+**	**+**	**+**	**+**	**+**
**PEX2**	**+**	**+**	**+**	**+**	**+**	**+**	**+**
**PEX3**	**+**	**+**	**+**	**+**	**+**	**+**	**+**
**PEX4**	**+**	**+**	**+**	**+**	**+**	**+**	**+**
**PEX5**	**+**	**+**	**+**	**+**	**+**	**+**	**+**
**PEX6**	**+**	**+**	**+**	**+**	**+**	**+**	**+**
**PEX7**	**+**	**+**	**+**	**+**	**+**	**+**	**+**
**PEX10**	**+**	**+**	**+**	**+**	**+**	**+**	**+**
**PEX11a**	**+**	**+**	**+**	**+**	**+**	**+**	**+**
**PEX11b**	**+**	**+**	**+**	**+**	**+**	**+**	**+**
**PEX11c**	**+**	**+**	**+**	**nf**	**nf**	**nf**	**nf**
**PEX12**	**+**	**+**	**+**	**+**	**+**	**+**	**+**
**PEX13**	**+**	**+**	**+**	**+**	**nf**	**nf**	**nf**
**PEX14**	**+**	**+**	**+**	**+**	**nf**	**nf**	**nf**
**PEX16**	**+**	**+**	**+**	**+**	**+**	**+**	**+**
**PEX19**	**+**	**+**	**+**	**+**	**+**	**+**	**+**
**PEX22**	**+**	**+**	**+**	**+**	**+**	**+**	**+**
**PEX26**	**+**	**+**	**cn**	**cn**	**nf**	**nf**	**nf**
**Catalase**	**+, PTS1**	**+, PTS1**	**+, PTS1**	**+, PTS1**	**+**	**+, PTS1**	**+, PTS1**

Note.—The presence (+) or absence (nf, not found) of the different putative peroxins identified in the Amoebozoans and Heteroloboseans included in our study is shown. Predicted sequences whose identity needs to be confirmed are specified (cn, confirmation needed). The different members of the Pex11 family found in these amoebae are labeled as Pex11a, Pex11b, and Pex11c. The presence (+) of at least one putative catalase sequence is also indicated. PTS1, peroxisomal targeting signal 1. Details regarding taxonomic classification can be found in Adl et al. (2019).

The protein sequences of the putative *A. castellanii* and *N. fowleri* peroxin homologues were retrieved directly from AmoebaDB. For *A. polyphaga*, *A. royreba*, *B. mandrillaris* and *N. lovaniensis*, the GeneWise algorithm (Birney et al. 2004) from the European Bioinformatics Institute (https://www.ebi.ac.uk/Tools/psa/genewise/; last accessed July 1, 2020) was used to obtain the protein sequence for each predicted peroxin. Using this algorithm, we compared the sequence of the query protein with the scaffold where the TBlastN analysis had identified the presence of the putative homologue. For sequences predicted to be located on the reverse strand, the reverse complement sequence of the scaffold was obtained before using it as input for the comparison.

To validate the BLAST hits, candidate sequences with *E-*values ≤1e-04 were subjected to reciprocal BlastP searches (https://blast.ncbi.nlm.nih.gov/Blast.cgi?PROGRAM=blastp&PAGE_TYPE=BlastSearch; BlastP 2.8.1+; last accessed July 1, 2020) against the query protein database (*H. sapiens*, *N. gruberi*, *A. castellanii*, *N. fowleri*, or *D. discoideum*). The top reciprocal hit that matched the original query with an *E-*value ≤1e-04 was considered as a putative homologue. To further confirm the identity of the homologues, their conserved domains were identified using NCBI’s Conserved Domain Search (https://structure.ncbi.nlm.nih.gov/cdd/wrpsb.cgi; last accessed July 1, 2020) (Marchler-Bauer and Bryant 2004).The presence of the Pex22 domain was determined using InterProScan 5 (http://www.ebi.ac.uk/interpro/search/sequence-search; last accessed July 1, 2020) (Jones et al. 2014). Unless otherwise indicated, all analyses were performed using the default parameters.

EMBOSS Needle (https://www.ebi.ac.uk/Tools/psa/emboss_needle/; last accessed July 1, 2020) (Needleman and Wunsch 1970) was used to determine the amino acid identity between the different members of the Pex11 family found in the Amoebozoans and Heteroloboseans included in this study (supplementary text 1, Supplementary Material online).

Putative catalase homologues were identified as described above. These sequences were then analyzed with CELLO2GO (http://cello.life.nctu.edu.tw/cello2go/; last accessed July 1, 2020) (Yu et al. 2014), DeepLoc-1.0 (https://services.healthtech.dtu.dk/service.php?DeepLoc-1.0; last accessed July 1, 2020) (Almagro Armenteros et al. 2017), PProwler 1.2 (http://bioinf.scmb.uq.edu.au:8080/pprowler_webapp_1-2/; last accessed July 1, 2020) (Hawkins and Bodén 2006), PTS1 predictor (http://mendel.imp.ac.at/pts1/; last accessed July 1, 2020) (Neuberger et al. 2003), Subcons (http://subcons.bioinfo.se/; last accessed July 1, 2020) (Salvatore et al. 2018), and SignalP-5.0 (https://services.healthtech.dtu.dk/service.php?SignalP-5.0; last accessed July 1, 2020) (Almagro Armenteros et al. 2019) to identify potential peroxisomal targeting sequences and to predict subcellular protein localizations. Potential transmembrane domains were detected with TMHMM v2.0 (https://services.healthtech.dtu.dk/service.php?TMHMM-2.0; last accessed July 1, 2020) (Krogh et al. 2001) and TOPCONS 2.0 (http://topcons.cbr.su.se/; last accessed July 1, 2020) (Tsirigos et al. 2015). Proteins were considered to have a peroxisomal location if they were not predicted to contain transmembrane domains and if not more than one of the subcellular location predictions differed from the others (supplementary table 9, Supplementary Material online) (Moog et al. 2017).

### Assessment of Genomic Completeness

The *A. castellanii* (WGS project AHJI01; https://www.ncbi.nlm.nih.gov/assembly/GCF_000313135.1; last accessed July 1, 2020), *A. polyphaga* (WGS project CDFK01; https://www.ncbi.nlm.nih.gov/assembly/GCA_000826345.1; last accessed July 1, 2020 and WGS project LQHA01; https://www.ncbi.nlm.nih.gov/assembly/GCA_001567625.1; last accessed July 1, 2020), *A. royreba* (WGS project CDEZ01; https://www.ncbi.nlm.nih.gov/assembly/GCA_000826365.1; last accessed July 1, 2020), *B. mandrillaris* (WGS project LFUI01; https://www.ncbi.nlm.nih.gov/assembly/GCA_001185145.1; last accessed July 1, 2020 and WGS project LEOU01; https://www.ncbi.nlm.nih.gov/assembly/GCA_001262475.1; last accessed July 1, 2020), *N. gruberi* (WGS project ACER01; https://www.ncbi.nlm.nih.gov/assembly/GCF_000004985.1; last accessed July 1, 2020), *N. fowleri* (WGS project AWXF01; https://www.ncbi.nlm.nih.gov/assembly/GCA_000499105.1; last accessed July 1, 2020), and *N. lovaniensis* (WGS project PYSW01; https://www.ncbi.nlm.nih.gov/assembly/GCA_003324165.1; last accessed July 1, 2020) genome assemblies were analyzed using the Benchmarking Universal Single-Copy Orthologs (BUSCO) v3.0 software (Waterhouse et al. 2018). The completeness of the different genome assemblies was assessed using the Eukaryota data set (https://busco-archive.ezlab.org/v3/; last accessed July 1, 2020), which contains 303 single-copy orthologs.

### Phylogenetic Analyses

For the phylogenetic analyses, a data set of protein sequences from various protozoans was assembled by performing BlastP searches against the NCBI and the EuPathDB (https://eupathdb.org/eupathdb/; release 46; last accessed July 1, 2020) (Aurrecoechea et al. 2017) databases, using the *A. castellanii* and *N. gruberi* Pex1 and Pex5 sequences as queries for the analyses. To avoid losing distant homologues, sequences from protozoans with *E*-values ≤0.05 were included in the data set. The sequences were then analyzed with NCBI’s Conserved Domain Search to verify that they contained the expected domains. Sequences were aligned with ProbCons 1.12 (Do et al. 2005) (http://www.phylogeny.fr/one_task.cgi?task_type=probcons; last accessed July 1, 2020) using the default parameters. Alignments were trimmed with BMGE 1.12.1 (Criscuolo and Gribaldo 2010), which is available on the NGPhylogeny platform (https://ngphylogeny.fr/; last accessed July 1, 2020) (Lemoine et al. 2019) using the default parameters (BLOSUM62 matrix; minimum block size of 5). RAxML-HPC2 8.2.12 (Stamatakis 2014) was used to perform maximum likelihood analyses using the protein GAMMA model and the LG amino acid substitution matrix (Le and Gascuel 2008). Bootstrap analyses (1,000 replicates) were performed to estimate branch support. These analyses were run using the CIPRES Science Gateway v3.3 (https://www.phylo.org/portal2/login!input.action; last accessed July 1, 2020) (Miller et al. 2010). The resulting trees were visualized using iTOL 5.3 (https://itol.embl.de/; last accessed July 1, 2020) (Letunic and Bork 2019) and rooted midpoint.

## Results

Of the numerous species of free-living amoebae described to date, only *Naegleria fowleri* (Heterolobosea), *Balamuthia mandrillaris*, *Sappinia pedata* and several *Acanthamoeba* species (Amoebozoa) are known to cause fatal infections of the CNS in humans (Visvesvara et al. 2007; Visvesvara 2013). In most of these amoebae, peroxisomes have not been described, possibly because of their variable size (0.1–1.0 μm) and scarce number. Thus, to determine if peroxisomes are present in three of these four genera of pathogenic free-living amoebae, we first performed morphological and cytochemical observations of trophozoites by transmission electron microscopy.

**Fig. 1:**
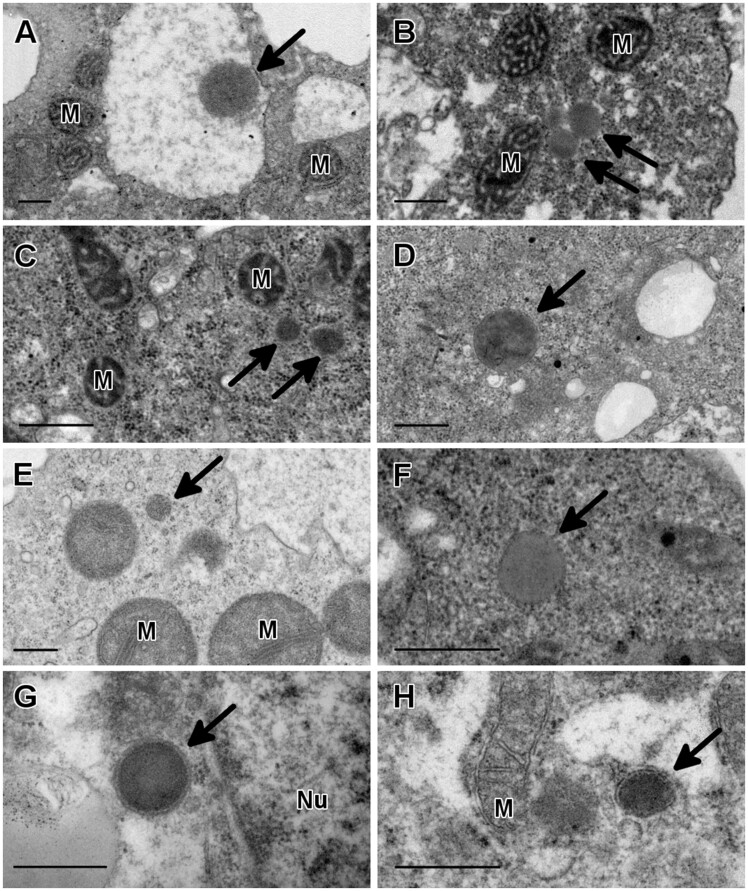
Identification of peroxisomes in three genera of pathogenic free-living amoebae by transmission electron microscopy. Spherical structures with a dark granular content and limited by a single membrane were observed in *Acanthamoeba griffini* (*A*), *Acanthamoeba polyphaga* (*B*), *Acanthamoeba royreba* (*C*), *Acanthamoeba castellanii* (*D*), *Balamuthia mandrillaris* (*E*), and *Naegleria fowleri* (*F*). These structures ranged in size from 0.2 to 0.9 µm and they were morphologically similar to peroxisomes from mouse liver samples, which are shown for comparison (*G*, *H*). M, mitochondrion; Nu, nucleus. Bar = 0.5 µm.

Round structures limited by a single membrane and varying in size from 0.3 to 0.6 µm were observed in thin sections of *A. castellanii*, *B. mandrillaris*, and *N. fowleri* (fig. 1*D*–*F*). These structures had a dark granular content similar to the one found in mouse liver control samples, which were used as positive controls for the identification of peroxisomes (fig. 1*G* and *H*). These spherical, single membrane structures were also observed in *A. griffini*, *A. polyphaga*, and *A. royreba* (fig. 1*A*–*C*), ranging in size from 0.15 to 0.9 µm.

**Fig. 2:**
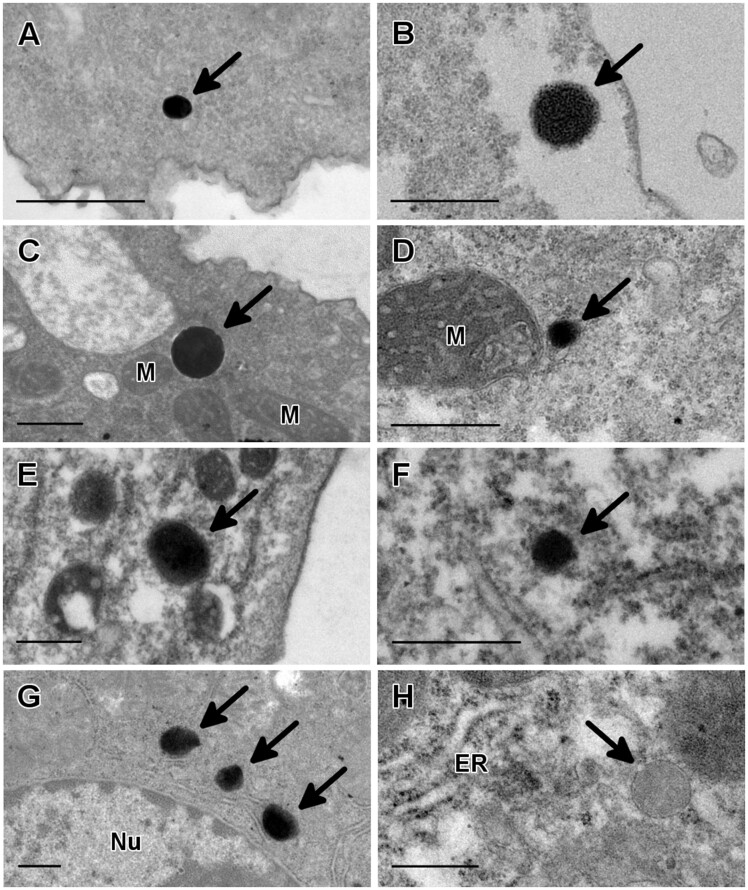
Peroxisome identification in three genera of pathogenic free-living amoebae by cytochemical staining. Thin sections of trophozoites were treated with diaminobenzidine and hydrogen peroxide to detect catalase activity. Positive reaction products were clearly seen in *Acanthamoeba griffini* (*A*), *Acanthamoeba polyphaga* (*B*), *Acanthamoeba royreba* (*C*), *Acanthamoeba castellanii* (*D*), *Balamuthia mandrillaris* (*E*) and *Naegleria fowleri* (*F*). In all of these amoebae, the labeling was deposited in round structures with a uniform electrondense content. Mouse liver samples were used as a positive control for the cytochemical staining (*G*). As a negative control for the cytochemical reaction, a mouse liver sample was incubated without H_2_O_2_ (*H*). ER, endoplasmic reticulum; M, mitochondrion; Nu, nucleus. Bar = 0.5 µm.

Next, trophozoites were treated with alkaline DAB and hydrogen peroxide to detect catalase activity. Examination of thin sections by transmission electron microscopy revealed the presence of distinct round particles measuring between 0.12 and 0.4 µm which showed positive reaction products for this peroxisome-specific cytochemical staining (Baumgart et al. 2003). These uniformly stained particles were clearly observed in *A. castellanii*, *B. mandrillaris*, and *N. fowleri*, but the number of peroxisomes per cell was low (fig. 2*D*–*F*). In other *Acanthamoeba* species incubated with DAB and H_2_O_2_ (*A. griffini*, *A. polyphaga*, and *A. royreba*), these structures were also clearly observed, varying only in size (0.1–0.4 µm) and in electron density (fig. 2*A*–*C*). Importantly, labeling was dependent on the presence of hydrogen peroxide, as no electrondense deposits were observed when control samples were incubated without this reagent (fig. 2*H*). The positive reaction products from this cytochemical staining strongly suggest that peroxisomes are present in *N. fowleri*, *B. mandrillaris*, and in various *Acanthamoeba* species.

Several authors have suggested that the identification of a number of peroxins in the genome of a given organism can be indicative of the presence of peroxisomes, as these proteins are present in all peroxisomes despite their metabolic content (Gabaldón 2010, 2018). Therefore, to support our previous morphological and cytochemical observations, we performed systematic comparative genomic analyses to determine if orthologues of different peroxin proteins were present in the genomes of *A. castellanii* (Clarke et al. 2013), *A. polyphaga* (Karlyshev 2019), *A. royreba*, *B. mandrillaris* (Detering et al. 2015; Greninger et al. 2015), *N. fowleri* (Zysset-Burri et al. 2014) and *N. lovaniensis* (Liechti et al. 2018).

As shown in table 1 and in the supplementary tables 2–8, Supplementary Material online, a complete set of peroxins is predicted to be present in the genomes of these free-living amoebae.

The sequences of the 15 putative peroxin homologues found in *A. castellanii* were identified by using the corresponding *H. sapiens* and *N. gruberi* protein sequences as queries for BlastP analyses against the *A. castellanii* protein database stored in AmoebaDB (table 1 and supplementary table 2, Supplementary Material online). All of these putative peroxin sequences were identified with the human queries (except for Pex4 and Pex22, which are not present in humans) and with the *N. gruberi* queries (except for Pex2, Pex13, Pex14, and Pex16, which have not been described in this amoeba). We also identified three distinct members of the Pex11 family (labeled Pex11a, Pex11b, and Pex11c) using the human queries and the presence of two of them was confirmed with the *N. gruberi* query. At the amino acid level, Pex11a was 20% identical to Pex11b but only 3.5% identical to Pex11c, whereas Pex11b and Pex11c shared a 4% identity at the protein level (supplementary text 1, Supplementary Material online). Interestingly, we also found a potential Pex26 homologue in *A. castellanii*. Although the BlastP and the reciprocal BlastP *E*-values were not significant, we did find the presence of the Pex26 domain in this sequence (supplementary table 2, Supplementary Material online).

The draft genomes of *A. polyphaga* and *A. royreba* are available on the NCBI WGS database as part of the *Phylogenomics of Acanthamoeba species* project (accession PRJEB7687). There are two draft genomes for *A. polyphaga* (CDFK00000000.1 and LQHA00000000.1) and one draft genome for *A. royreba* (CDEZ00000000.1). By performing TBlastN searches using the protein sequences of the peroxins identified in *A. castellanii* as queries, we identified 15 putative peroxin homologues in these amoebae (table 1 and supplementary tables 3 and 4, Supplementary Material online). Similarly to *A. castellanii*, we found three distinct members of the Pex11 family (also labeled as Pex11a, Pex11b, and Pex11c) in these organisms. For *A. polyphaga*, Pex11a is 20% identical to Pex11b and 5% identical to Pex11c, whereas Pex11b and Pex11c share a 6% identity at the amino acid level (supplementary text 1, Supplementary Material online). For *A. royreba*, the amino acid identity between Pex11a, and Pex11b, and Pex11c is 19% and 23%, respectively, whereas the predicted Pex11b and Pex11c protein sequences are 20% identical (supplementary text 1, Supplementary Material online).

In contrast to *A. castellanii*, we found more than one putative homologue for most peroxins in both of these *Acanthamoeba* species. For *A. polyphaga*, we found two or more putative peroxin homologues in either the CDFK or the LQHA assemblies, with the exception of Pex2, Pex10, Pex13, and Pex14, which only have one predicted homologue in each assembly (supplementary table 3, Supplementary Material online). All of these putative homologues have the expected functional domains except for the Pex12 sequences, which have either the mRING_PEX12 domain or the PEX2_PEX12 domain. To determine if this could be due to a high level of fragmentation of the *A. polyphaga* assemblies, we examined the putative Pex12 sequences and we found that one of them was located in a very short contig (2,250 nt) and the rest were located in the terminal part of their respective contigs, which explains why we were unable to find a putative Pex12 sequence with both domains. Importantly, when the sequences bearing the different PEX12 domains are aligned, they appear to be part of the same sequence (supplementary text 2, Supplementary Material online).

Similarly, we also found more than one putative homologue for each peroxin in *A. royreba*, except for Pex2, Pex7, Pex19, and Pex22 (supplementary table 4, Supplementary Material online). Additionally, we found various potential Pex26 homologues in these organisms (supplementary tables 3 and 4, Supplementary Material online), but since we could only identify the corresponding domain in one of the sequences from *A. polyphaga* (supplementary table 3, Supplementary Material online), further studies are necessary to confirm that these sequences are indeed Pex26 homologues. Overall, our bioinformatic results, together with the previous ultrastructural and cytochemical observations made in this study, indicate that peroxisomes are also present in *A. polyphaga* and *A. royreba*.

To determine whether the presence of more than one sequence for most of the putative peroxins identified in *A. polyphaga* and *A. royreba* could be due to problems during the assembly procedure, we evaluated the quality of these genome assemblies using the Benchmarking Universal Single-Copy Orthologs (BUSCO) assessment tool, which measures assembly completeness based on expected gene content. BUSCOs are expected to be found as single-copy orthologs and duplication of these genes is considered as a rare event (Waterhouse et al. 2018). When the assemblies were analyzed using the 303 single-copy orthologs from the eukaryotic data set, only six duplicated BUSCOs were found in the *A. polyphaga* LQHA assembly, whereas 66 duplicated BUSCOs were found in the *A. polyphaga* CDFK assembly (supplementary fig. 1, Supplementary Material online). Similarly, 132 duplicated BUSCOs were found in the *A. royreba* CDEZ assembly. The large number of duplicated BUSCOs found in *A. royreba* and in the *A. polyphaga* CDFK assembly suggests that the genome assembly procedure could have failed to collapse the sequenced haplotypes (Waterhouse et al. 2018).

Next, we performed in silico analyses to identify putative peroxin homologues in *B. mandrillaris.* There are two genome assemblies from different *B. mandrillaris* strains deposited in the NCBI WGS database, one corresponding to the CDC-V039 isolate under accession number LFUI00000000.1 (Detering et al. 2015) and another corresponding to the 2046 strain under accession number LEOU00000000.1 (Greninger et al. 2015). Neither genome assembly has been fully annotated, thus, we performed TBlastN searches against both *B. mandrillaris* draft genomes using the protein sequences of various peroxins from *H. sapiens* and *N. gruberi* as queries.

As shown in table 1 and in the supplementary table 5, Supplementary Material online, 15 putative peroxins were identified in both *B. mandrillaris* genome assemblies. The conserved domains specific to each peroxin were also predicted to be present in the proteins identified in *B. mandrillaris*, and the results of the reciprocal BlastP analyses against the query proteomes confirmed the identity of the putative homologues.

In contrast to *A. castellanii*, only Pex5, Pex6, Pex7, Pex10, and Pex12 were identified in *B. mandrillaris* with both the human and the *N. gruberi* protein sequences. Pex1 and Pex3 were only identified with the human queries, whereas Pex11 and Pex19 were only identified with the *N. gruberi* queries. Pex2 and Pex16 were also identified with the human queries, but we were unable to identify putative homologues for Pex13 or Pex14 when the respective human sequences were used as queries for the TBlastN analyses. Similarly, Pex4 was identified with the corresponding *N. gruberi* protein sequence, but not Pex22.

Because the human and the *N. gruberi* queries appeared to be too divergent from *B. mandrillaris* to detect peroxin homologues with confidence, we decided to confirm our results by using the sequences of the peroxins identified in *A. castellanii*, which is more closely related to *B. mandrillaris*.

When the *A. castellanii* sequences were used as queries, we were able to confirm the presence of all of the putative peroxins identified with either the human queries, the *N. gruberi* queries, or both. Additionally, the *A. castellanii* sequences allowed us to identify putative homologues for Pex13 and Pex14—which had not been found with the human queries—, a putative Pex22 homologue—which had not been identified with the *N. gruberi* query—and a second member of the Pex11 family, which had not been identified with either query. We identified two sequences for Pex1, Pex7, Pex12, and Pex13 in the LFUI release, whereas two Pex14 sequences were identified in the LEOU release. For Pex11, we found two different sequences in the LFUI release, which represent distinct members of this family (table 1 and supplementary table 5, Supplementary Material online), as they are only 15% identical at the protein level (supplementary text 1, Supplementary Material online). We also found three potential Pex26 homologues, but given that the sequences were very short, we could not confirm the presence of the Pex26 domain. The fact that only the *A. castellanii* queries allowed us to identify all of the putative peroxin sequences in *B. mandrillaris* underscores the importance of using closely related sequences to confirm the presence or absence of putative homologues.

In contrast to *B. mandrillaris* and the different *Acanthamoeba* species, only 13 putative peroxins were identified in *N. fowleri*, as Pex13 and Pex14 were not found in this amoeba (table 1 and supplementary table 6, Supplementary Material online). The putative peroxin sequences were identified by using the corresponding *H. sapiens* and *N. gruberi* protein sequences as queries for BlastP analyses against the *N. fowleri* protein database stored in AmoebaDB. All these sequences were identified with the human queries (except for Pex4 and Pex22, which are absent in humans) and with the *N. gruberi* queries (except for Pex2 and Pex16, which have not been reported in this amoeba). Intriguingly, a longer version of the Pex2 sequence than the one reported in AmoebaDB was identified when the *D. discoideum* Pex2 sequence was used as query for TBlastN analyses against the *N. fowleri* NCBI WGS database. This longer sequence includes both the PEX2_PEX12 and the RING-HC_PEX2 domains (supplementary table 6, Supplementary Material online). Finally, in contrast to *N. gruberi*—in which only one member of the Pex11 family has been described—two putative members of this family (labeled Pex11a and Pex11b) were identified in *N. fowleri*. Interestingly, both Pex11 sequences were identified with the *N. gruberi* query but only one of them was found with the human queries. These putative Pex11a and Pex11b sequences are 19% identical at the amino acid level (supplementary text 1, Supplementary Material online).

Recently, Liechti et al. (2018) characterized the genome of *N. lovaniensis* which, of the 47 species of *Naegleria* described to date, is the one that is most closely related to the pathogenic *N. fowleri* (De Jonckheere 2014). Using the peroxin sequences from *D. discoideum*, *N. gruberi*, and *N. fowleri* as queries for TBlastN searches against the *N. lovaniensis* WGS database, we identified 13 putative peroxin homologues in this amoeba (table 1 and supplementary table 7, Supplementary Material online). All of these putative peroxin sequences were identified with the *D. discoideum* queries (except for Pex3, Pex16, and Pex19), the *N. gruberi* queries (except for Pex2 and Pex16, which have not been described in this amoeba) and with the *N. fowleri* queries. Importantly, the putative Pex16 homologue, which was not found with the *D. discoideum* query, was identified when the *N. fowleri* Pex16 sequence was used as a query for the TBlastN analysis. All of the putative peroxin homologues detected in *N. lovaniensis* have the expected functional domains and, similarly to *N. fowleri*, two putative members of the Pex11 family were also identified in this amoeba, which are 20% identical at the amino acid level (supplementary text 1, Supplementary Material online). Also, as observed in *N. fowleri*, no Pex13 or Pex14 homologues were found in *N. lovaniensis*.

The fact that *N. fowleri* and *N. lovaniensis* had the same number of putative peroxin homologues prompted us to check the *N. gruberi* genome for sequences coding for Pex2, Pex16, and for another member of the Pex11 family, which had not been reported in this amoeba thus far. Using the corresponding *N. fowleri* sequences as queries for TBlastN analyses against the *N. gruberi* NCBI WGS database, we were able to identify putative homologues for these peroxins, and we also found longer versions of Pex1 and Pex6 (table 1 and supplementary table 8, Supplementary Material online). All of these putative peroxins have the expected functional domains and the reciprocal BlastP analyses against the query database confirmed the homology between the sequences. These results constitute the first report on the presence of Pex2 and Pex16 in *N. gruberi*. As for the two members of the Pex11 family, one of them (ACER01000200.1) has not been reported previously, whereas the second one (ACER01000043.1) has been described as part of the *N. gruberi* proteome (UniProt ID: D2V0G7). When compared at the amino acid level, these two putative members of the Pex11 family are 22% identical (supplementary text 1, Supplementary Material online). As expected, we were unable to identify homologues for Pex13 or Pex14 in this amoeba, which suggests that they are missing in this genus.

Based on the results of our cytochemical assays, we then performed BLAST analyses to confirm the presence of catalase in the different Amoebozoans and Heteroloboseans included in our study. As expected, we were able to identify several putative homologues of this enzyme in all of these organisms (table 1 and supplementary tables 2–7, Supplementary Material online). Our in silico analyses also predicted the presence of a peroxisomal targeting sequence (PTS1) in at least one of the putative catalase enzymes found in each of the different Amoebozoans included in our study (table 1 and supplementary table 9, Supplementary Material online). For *N. fowleri* and *N. lovaniesis*, one of the two putative catalase sequences identified in these amoebae was predicted to have a PTS1 signal, but none of the putative catalase sequences from *N. gruberi* was predicted to contain this peroxisomal targeting sequence (table 1 and supplementary table 9, Supplementary Material online).

**Fig. 3:**
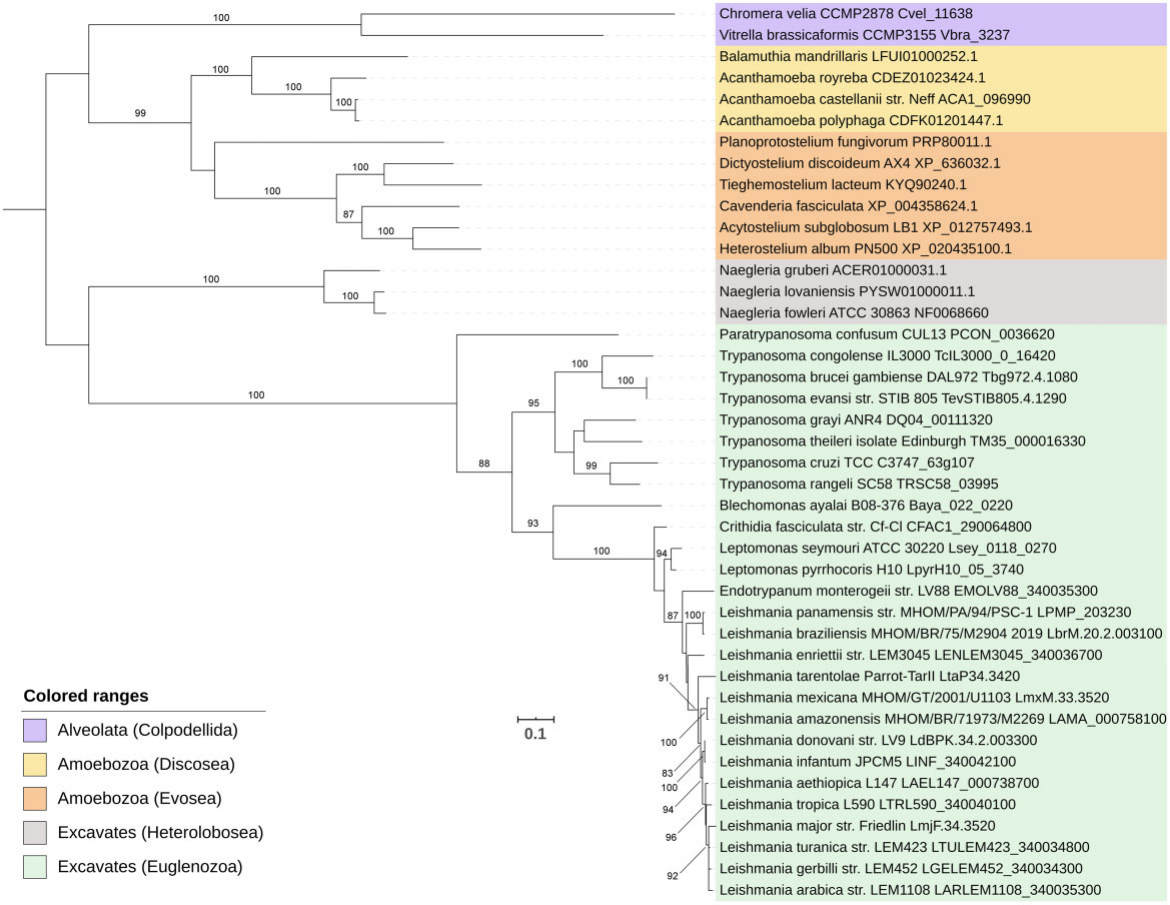
Phylogenetic analysis of the peroxisomal biogenesis protein Pex1. The consensus tree was obtained with RAxML-HPC2 8.2.12. Bootstrap values >80% are indicated. The tree is rooted midpoint and the scale bar indicates the mean number of amino acid substitutions per site. Sequences are colored to denote the different taxonomic lineages (see box).

**Fig. 4:**
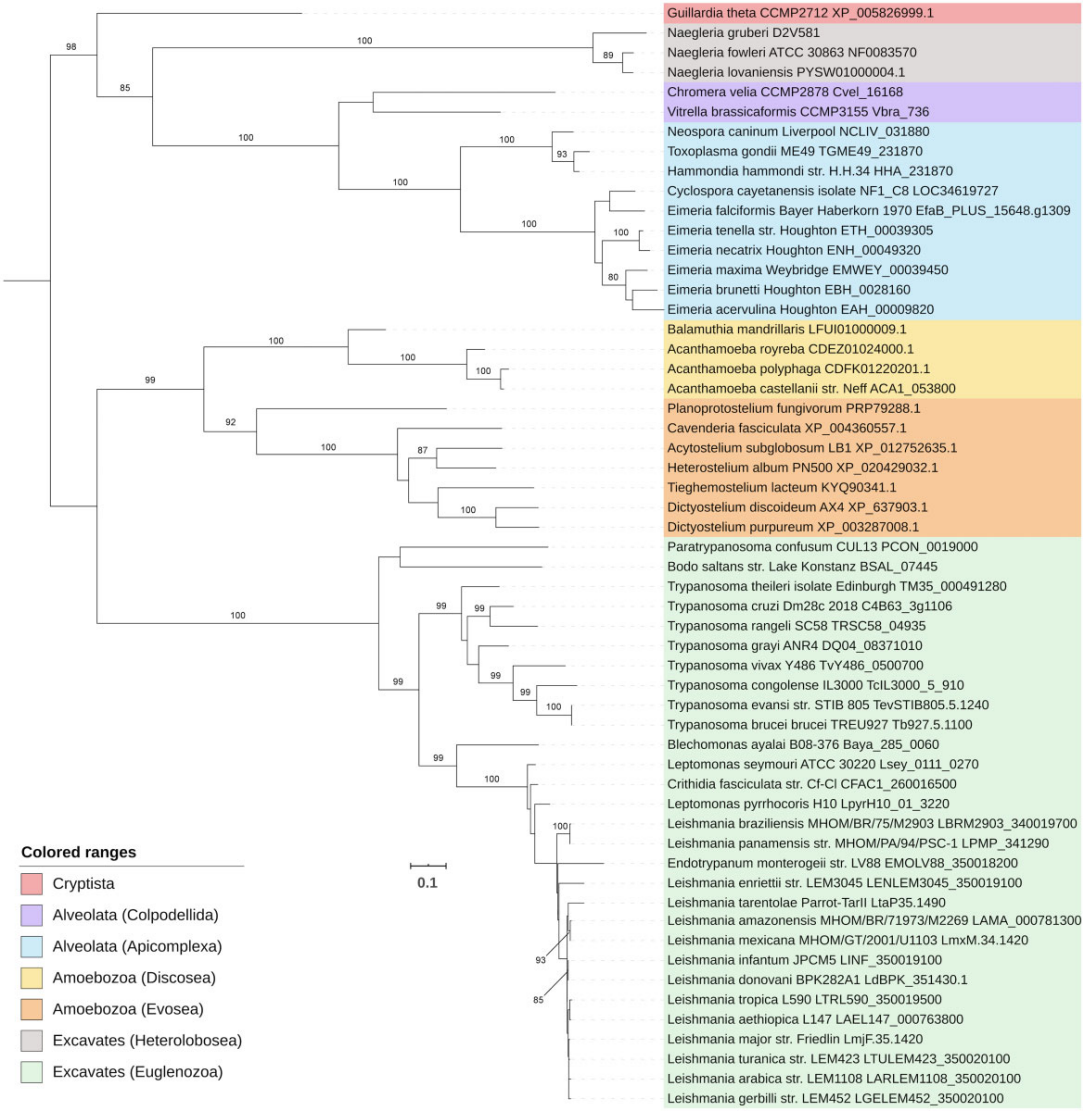
Phylogenetic analysis of the peroxisomal biogenesis protein Pex5. RAxML-HPC2 8.2.12 was used to obtain the consensus tree. Bootstrap values >80% are shown. The tree is midpoint rooted and the scale bar shows the mean number of amino acid substitutions per site. Sequences are colored to indicate their taxonomic lineage (see box).

Finally, we performed two phylogenetic analyses using the ATPase Pex1 and Pex5, the receptor for the PTS1 protein import pathway. The phylogeny of these peroxins suggests that *A. castellanii* is more closely related to *A. polyphaga* than to *A. royreba*. In both phylogenies, this grouping had a high statistical support. These organisms were then grouped with *B. mandrillaris* and this cluster was then grouped with the other Amoebozoans included in the phylogenetic analyses (figs. 3 and 4). Similarly, the three *Naegleria* species included in our study were grouped in a distinct clade; however, this cluster was only grouped with the other Excavates in the Pex1 phylogeny (fig. 3). In both phylogenetic analyses, the pathogenic *N. fowleri* was found to be more closely related to *N. lovaniensis* than to *N. gruberi* (figs. 3 and 4).

## Discussion

Peroxisomes are metabolically diverse organelles that are present in most eukaryotic cells. The interest in peroxisomes and in their physiological functions has increased significantly since numerous studies have demonstrated their active participation in crucial metabolic processes such as the β-oxidation of fatty acids, the biosynthesis of ether phospholipids, and the metabolism of reactive oxygen species. These organelles have also been shown to rapidly assemble, multiply, and degrade in response to metabolic needs (Islinger et al. 2012). The importance of peroxisomes in maintaining cellular homeostasis is also highlighted by the fact that defects in proteins involved in the biogenesis of peroxisomes or in single peroxisomal enzymes can result in inherited peroxisomal disorders (Platta and Erdmann 2007).

Although peroxisomes are involved in numerous cellular processes, some protists can survive without them, including Apicomplexan parasites such as *Plasmodium* and *Cryptosporidium* (Žárský and Tachezy 2015) and parasites which lack canonical mitochondria—but which contain mitochondrion-related organelles—such as *Entamoeba histolytica*, *Giardia lamblia*, and *Trichomonas vaginalis* (Makiuchi and Nozaki 2014; Žárský and Tachezy 2015). Nonetheless, peroxisomes have been observed in other species of protozoa including *Tetrahymena pyriformis*, *Paramecium caudatum*, *Euglena gracilis*, *D. discoideum* as well as in *Trypanosoma* and *Leishmania* among others (Müller 1975; Gabaldón 2010; Gabaldón et al. 2016). Despite this remarkable diversity regarding the presence or absence of peroxisomes in free-living and parasitic protists, many protozoan lineages remain understudied. In this respect, information regarding the presence of peroxisomes in opportunistic free-living amoebae is limited and in many cases it is still unknown if these organisms have peroxisomes at all.

By transmission electron microscopy, various round structures with a dark granular content and surrounded by a single membrane were observed in several *Acanthamoeba* species, as well as in *B. mandrillaris* and *N. fowleri* (fig. 1*A*–*F*). Our cytochemical observations indicated that these structures contained catalase, as positive reaction products were observed when samples were incubated with DAB and hydrogen peroxide (fig. 2*A*–*F*). DAB labeling has been used extensively to detect peroxisomes by light and electron microscopy and its widespread use has demonstrated the ubiquity of this organelle in different organisms (Fahimi and Baumgart 1999; Baumgart et al. 2003). Thus, the presence of positive reaction products for this cytochemical staining, together with our ultrastructural observations, provides experimental evidence for the existence of peroxisomes in these free-living amoebae. To the best of our knowledge, our ultrastructural observations in *N. fowleri* constitute the first morphological and cytochemical evidence for the presence of peroxisomes in a member of the Heterolobosea (Gabaldón et al. 2016). Moreover, these results represent the first morphological and cytochemical evidence for the presence of peroxisomes in *B. mandrillaris* as well as in *A. polyphaga*, *A. royreba*, and *A. griffini*.

Our ultrastructural observations in the different *Acanthamoeba* species are complemented by earlier biochemical studies which found that peroxisomes are present in this genus. Childs (1973a, 1973b) reported the presence of peroxisomes in *Hartmannella culbertsoni* (now known as *Acanthamoeba culbertsoni*) by using DAB and hydrogen peroxide for catalase localization, whereas Müller and MØller (1969) were the first to describe the presence of peroxisome-like particles containing urate oxidase and catalase in a free-living amoeba by isopycnic centrifugation of *Acanthamoeba* trophozoites. These enzymes were also found in the slime mold *D. discoideum* (Parish 1975; Hayashi and Suga 1978) which, together with *Acanthamoeba*, was one of the first members of the Amoebozoa in which the presence of peroxisomes was described by biochemical assays.

In recent years, the genome sequences of numerous organisms from different taxonomic groups have become available, allowing the use of comparative genomics to identify putative peroxisomal proteins based on a high degree of sequence similarity (Wanders and Waterham 2006; Gabaldón 2018). Because peroxins are common to all peroxisomes despite their enzymatic content, their genomic identification can be considered as an indicator for the existence of peroxisomes in a particular organism (Gabaldón 2018). This bioinformatic approach has provided compelling evidence regarding the presence or absence of this organelle in different organisms, including several protist lineages (Moog et al. 2017; Ludewig-Klingner et al. 2018).

Our comparative genomic analyses allowed us to identify a group of 15 putative peroxins in the Amoebozoans *A. castellanii*, *A. polyphaga*, *A. royreba*, and *B. mandrillaris* (table 1). Among these putative peroxins, we identified Pex5, which is a cytosolic receptor for proteins destined to the peroxisome that bear the peroxisomal targeting signal type 1 (PTS1) and Pex7, which is a soluble receptor for proteins that carry the PTS2 sequence. We also found putative homologues for Pex13 and Pex14, which are membrane proteins that form a docking complex that interacts with the receptors to translocate their cargo proteins into the peroxisomal matrix. The ubiquitin conjugating enzyme Pex4 and its membrane anchor, Pex22, as well as the RING ubiquitin ligases Pex2, Pex10, and Pex12—which monoubiquitinate Pex5 for recycling or polyubiquitinate it for degradation in the proteasome—were also predicted to be present in these amoebae. Likewise, the AAA-type ATPases Pex1 and Pex6, which recycle the monoubiquitinated Pex5 back to the cytoplasm, were identified in these organisms. We also found putative homologues for Pex3, Pex16, and Pex19, which are involved in the insertion of membrane proteins into the peroxisomal membrane. Finally, several members of the Pex11 family, which is essential for peroxisome division, were also identified in these Amoebozoans (peroxins functions are described in Girzalsky et al. [2010], Kim and Hettema [2015], and Platta et al. [2016]). The fact that at least two distinct members of the Pex11 family were found in these Amoebozoans is not uncommon, as several Pex11 variants have been described in other organisms. *Arabidopsis thaliana* has five Pex11-related proteins (Lingard and Trelease 2006), whereas three distinct members of this family have been identified in mammals and filamentous fungi (Thoms and Erdmann 2005; Kiel et al. 2006). Besides being fundamental for peroxisome proliferation, Pex11 also regulates peroxisome morphology, number, and size (Schrader et al. 2012). Although their functions are partially redundant, the different members of this family appear to have some degree of functional variability: in plants, two Pex11 variants have been shown to increase the number of peroxisomes, whereas others induce elongation or peroxisome aggregation (Lingard and Trelease 2006; Schrader et al. 2012). It will be interesting to determine whether the different members of the Pex11 family identified in these Amoebozoans have overlapping or independent functions, as well as their specific role during peroxisome proliferation.

Besides these 15 putative peroxins, a possible homologue for Pex26 was also identified in these Amoebozoans. Pex26 is present in mammals but it is absent in plants as well as in *Saccharomyces cerevisiae* and related yeasts (Islinger et al. 2010). In mammals, Pex26 anchors the Pex1–Pex6 complex to the peroxisomal membrane (Matsumoto et al. 2003), a function that is performed by Pex15 in yeasts (Kalel and Erdmann 2018). The Pex26 domain was only identified in the putative *A. castellanii* homologue and in just one of the three putative homologues found in *A. polyphaga* (supplementary tables 2 and 3, Supplementary Material online). None of the potential Pex26 homologues described in *A. royreba* bears the corresponding domain, despite the fact that these sequences had high TBlastN and reciprocal BlastP *E*-values (supplementary table 4, Supplementary Material online). It is possible that the sequence for this domain has diverged significantly from that of other organisms, which would explain why we were unable to identify it. Alternatively, these putative homologues might have become redundant and may be evolving new functions or they may not be *bona fide* Pex26 homologues. Importantly, we could not identify Pex15 homologues in any of these amoebae. Overall, this group of predicted peroxins is probably sufficient for the generation and maintenance of functional peroxisomes in *A. castellanii*, *A. polyphaga*, *A. royreba*, and *B. mandrillaris*.

Our bioinformatic data regarding the presence of peroxins in *Acanthamoeba* and *B. mandrillaris* indicate that the peroxin content of these organisms is similar to that of other Amoebozoans, because all of the putative peroxins homologues found in *Acanthamoeba* and *B. mandrillaris*—with the exception of Pex22 and Pex26—have also been described in *D. discoideum* (supplementary table 1, Supplementary Material online). As more genomic data from other Amoebozoan lineages become available, future studies will provide additional insights regarding the peroxin content of this taxonomic group.

With the exception of Pex13 and Pex14, a complete set of putative peroxins was also identified in the pathogenic *N. fowleri* and in the closely related but nonpathogenic *N. lovaniensis* (table 1). Our bioinformatic analyses also found putative homologues for the previously unidentified Pex2, Pex16, and for a second member of the Pex11 family in the nonpathogenic *N. gruberi* (supplementary table 8, Supplementary Material online), confirming that all three *Naegleria* species share the same set of peroxins regardless of their pathogenic capacity. We also failed to identify a Pex26 homologue in these *Naegleria* species, which suggests that this peroxin might not be necessary for peroxisome function in these organisms. Thus, this group of Heteroloboseans appear to have a slightly reduced set of peroxins with respect to the Amoebozoans analyzed in this study.

The absence of Pex13 and Pex14 in *N. gruberi*, *N. fowleri*, and *N. lovaniensis* raises the question of whether these peroxins were present in *Naegleria* and were subsequently lost during evolution or whether these amoebae developed an import mechanism that does not require this particular docking complex. Typically, Pex13 and Pex14 form a membrane-bound protein complex that interacts with Pex5 and Pex7 to import proteins into the peroxisomal matrix. Given that Pex5 and Pex7 are present in all three *Naegleria* species, future studies should aim to understand how this import mechanism functions in the absence of the Pex13–Pex14 docking complex. Alternatively, the Pex13 and Pex14 sequences could have diverged significantly in these Heteroloboseans, to the point that they could not be detected by our in silico analyses. Importantly, the absence of Pex13 and Pex14 has been reported in other organisms that contain peroxisomes such as the diatoms *Thalassiosira pseudonana* (Schlüter et al. 2006) and *Phaeodactylum tricornutum* (González et al. 2011). However, these organisms also lack Pex7, which indicates that they have lost the PTS2 import pathway (González et al. 2011). Because all three *Naegleria* species are predicted to have Pex7 homologues and *N. gruberi* has been reported to have at least one peroxisomal protein with a predicted PTS2 signal (González et al. 2011), it seems unlikely that this import pathway is absent in *Naegleria*.

As shown in table 1, with the exception of Pex13 and Pex14, our comparative genomic results support previous observations which indicate that, of the more than 30 peroxins described to date, at least 13 of them (Pex1–3, 5–7, 10–14, 16, and 19) are present in all major eukaryotic lineages (Žárský and Tachezy 2015). Besides this core group of peroxins, we found putative homologues for Pex4 and Pex22 in all of the Amoebozoans and Heteroloboseans included in our study. Both of these peroxins are absent in mammals, but they are expressed in plants, filamentous fungi, and yeasts (Islinger et al. 2010; Platta et al. 2016).

In contrast to the other free-living amoebae analyzed in this study, two or more putative copies for almost every peroxin were found in *A. polyphaga* (supplementary table 3, Supplementary Material online) and *A. royreba* (supplementary table 4, Supplementary Material online). When the completeness of the genome assemblies used in this study was evaluated using the BUSCO software, we found that the *A. polyphaga* CDFK assembly, the *A. royreba* CDEZ assembly, and the *B. mandrillaris* LFUI assembly had a large number of duplicated BUSCOs compared with the other assemblies (supplementary fig. 1, Supplementary Material online). Although the precise ploidy levels of these Amoebozoans have not been determined, there are strong indications that they are polyploid (Matsunaga et al. 1998; Detering et al. 2015). Thus, the multiple gene copies that were found in these assemblies might be heterozygous alleles that the assembly procedure failed to collapse (Waterhouse et al. 2018). Nonetheless, additional studies such as genomic and expression analyses are needed to confirm that these putative copies are indeed sequence assembly artifacts.

To the best of our knowledge, none of the putative peroxins identified in this study has been previously reported in *A. polyphaga*, *A. royreba*, *B. mandrillaris*, or *N. lovaniensis*. In contrast, most of the putative peroxins described in *N. fowleri* are properly annotated in AmoebaDB or in the UniProt database, but most of the *A. castellanii* and *N. gruberi* peroxins are not annotated as such and instead they are classified according to their domains or as hypothetical proteins.

Our bioinformatic analyses also allowed us to confirm the presence of several putative catalase sequences in all of these organisms (table 1 and supplementary tables 2–7, Supplementary Material online). For each of these organisms, at least one putative catalase sequence was predicted to be located in the peroxisome, as they contain a PTS1 signal (supplementary table 9, Supplementary Material online). These results support our previous cytochemical observations which indicated that catalase was present in the peroxisomes of *A. castellanii, A. polyphaga, A. royreba*, *B. mandrillaris*, and *N. fowleri* (fig. 2). All of the putative catalase sequences from *B. mandrillaris* and from the different *Acanthamoeba* species had the same PTS1 signal (-AKL), which suggests that this sequence is conserved in these Amoebozoans. As for the Heteroloboseans analyzed in this study, only one putative catalase sequence from *N. fowleri* and one putative catalase sequence from *N. lovaniesis* were predicted to have a PTS1 signal (-NNL). The -NNL tripeptide has been identified as a putative PTS1 sequence in the sterol carrier protein 2 (SCP2) from the yeast *Yarrowia lipolytica* (Ferreyra et al. 2006). Although this tripeptide has not been experimentally validated as a functional PTS1, fungal SCP2 is strictly peroxisomal and it is believed to be involved in the peroxisomal oxidation of long-chain fatty acids (Ferreyra et al. 2006). In contrast to *N. fowleri* and *N. lovaniensis*, none of the putative catalase sequences reported in *N. gruberi* appears to have a PTS1, although one of them has a C-terminal tripeptide that is similar to the one identified in the other *Naegleria* species (-QNL). Importantly, Opperdoes et al. (2011) also found that the predicted *N. gruberi* catalase sequence lacks a PTS signal. Interestingly, the PTS1 sequence identified in *N. fowleri* and *N. lovaniensis* (-NNL) differs from the predicted PTS1 signals identified by Opperdoes et al. in other *N. gruberi* peroxisomal enzymes, which were found to be predominantly -SKL or a variant thereof (-NKL/-SKM).

It should be noted that the bioinformatic approach used for identifying peroxisomal homologues in these Amoebozoans and Heteroloboseans has some limitations. The putative protein sequences of the peroxins and catalases identified in *A. polyphaga*, *A. royreba*, *B. mandrillaris*, *N. gruberi*, and *N. lovaniensis* were determined using the GeneWise algorithm, which has been used both as a stand-alone tool and as part of a gene prediction pipeline (Birney et al. 2004). GeneWise identifies potentially homologous genes by aligning a genomic DNA sequence to a homologous protein sequence (Birney et al. 2004). This tool is able to identify complete genes when the homologues are very similar, but when the homologues are more distant, GeneWise is only able to detect part of the exon structure (Guigó et al. 2000; Yeh et al. 2001). In fact, a known disadvantage of this algorithm is that it fails to detect complete terminal exons, as GeneWise predictions are limited to sites where there is evidence of similarity (Birney et al. 2004). Most of the putative protein sequences identified in *A. polyphaga*, *A. royreba*, *N. lovaniensis*, and *N. gruberi* appear to be fairly complete, as these sequences have a high level of similarity to their respective queries. However, in some cases, GeneWise could only find fragments of these putative proteins, as observed for the *A. royreba* Pex22 sequence and for the *B. mandrillaris* Pex14, Pex16, Pex19, and Pex22 sequences (supplementary tables 4 and 5, Supplementary Material online). Despite being incomplete, all of these putative sequences bear the expected PEX domains, which suggests that the aligned region was limited to the conserved domains and that the queries and the putative proteins have a low level of similarity (Guigó et al. 2000). Thus, further analyses are needed to obtain the complete sequences of the putative peroxisomal proteins identified in our bioinformatic surveys. Likewise, as our studies were performed using genomic data, transcriptomic and proteomic studies are needed to confirm the expression of these putative peroxisomal proteins.

With respect to the phylogenetic analyses of Pex1 and Pex5, *A. castellanii* was found to be more closely related to *A. polyphaga* than to *A. royreba* (figs. 3 and 4). Importantly, a previous phylogenetic reconstruction based on the 18S ribosomal genes of various *Acanthamoeba* species also grouped *A. castellanii* with *A. polyphaga*, whereas *A. royreba* was clustered with other *Acanthamoeba* species (Chelkha et al. 2018). The *Acanthamoeba* cluster was then grouped with *B. mandrillaris*, confirming the close phylogenetic relationship between *Acanthamoeba* and *Balamuthia* (figs. 3 and 4). These results are also in agreement with previous phylogenetic analyses of the mitochondrial cox1 protein and the 28S rRNA gene, which also clustered these two genera together (Greninger et al. 2015). As for the Heteroloboseans included in our study, the pathogenic *N. fowleri* was found to be more closely related to *N. lovaniensis* than to *N. gruberi*, as reported in previous studies (De Jonckheere 2014; Liechti et al. 2018). Importantly, only the Pex1 phylogeny grouped the *Naegleria* cluster with the other Excavates included in the analysis (figs. 3 and 4).

In summary, our morphological, cytochemical, and genomic data demonstrate that peroxisomes are present in *B. mandrillaris*, *A. castellanii*, *A. polyphaga*, and *A. royreba*. Our cytochemical and morphological data also indicate that *A. griffini* has peroxisomes, but because the genome of this amoeba has not been sequenced, we could not determine if some or all of the peroxins and catalase homologues that were identified in the other *Acanthamoeba* species are also present in *A. griffini*.

Our ultrastructural, cytochemical, and comparative genomic data also indicate that *N. fowleri* has *bona fide* peroxisomes. Similarly, our comparative genomic results indicate that peroxisomes are also present in the nonpathogenic *N. lovaniensis*, although morphological observations are still needed to confirm the presence of this organelle. Additionally, it will be important to explore the enzymatic content of the peroxisomes of these Amoebozoans and Heteroloboseans to establish their precise role in their metabolism and to increase our understanding of the diversity of peroxisomal functions in different eukaryotic lineages.

## Supplementary Material

Supplementary data are available at *Genome Biology and Evolution* online.

## Supplementary Material

evaa129_Supplementary_Data

## References

[evaa129-B1] Adl SM, et al 2019. Revisions to the classification, nomenclature, and diversity of eukaryotes. J Eukaryot Microbiol. 66(1):4–119. 30257078 10.1111/jeu.12691 PMC6492006

[evaa129-B2] Aghajani A Dabirzadeh M Maroufi Y Hooshyar H. 2016. Identification of *Acanthamoeba* genotypes in pools and stagnant water in ponds in Sistan region in Southeast Iran. Turkiye Parazitol Derg. 40(3):132–136. 27905281 10.5152/tpd.2016.4428

[evaa129-B3] Almagro Armenteros JJ Sønderby CK Sønderby SK Nielsen H Winther O. 2017. DeepLoc: prediction of protein subcellular localization using deep learning. Bioinformatics 33(21):3387–3395. 29036616 10.1093/bioinformatics/btx431

[evaa129-B4] Almagro Armenteros JJ, et al 2019. SignalP 5.0 improves signal peptide predictions using deep neural networks. Nat Biotechnol. 37(4):420–423. 30778233 10.1038/s41587-019-0036-z

[evaa129-B5] Altschul SF Gish W Miller W Myers EW Lipman DJ. 1990. Basic local alignment search tool. J Mol Biol. 215(3):403–410. 2231712 10.1016/S0022-2836(05)80360-2

[evaa129-B6] Altschul SF, et al 1997. Gapped BLAST and PSI-BLAST: a new generation of protein database search programs. Nucleic Acids Res. 25(17):3389–3402. 9254694 10.1093/nar/25.17.3389 PMC146917

[evaa129-B7] Aurrecoechea C, et al 2011. AmoebaDB and MicrosporidiaDB: functional genomic resources for Amoebozoa and Microsporidia species. Nucleic Acids Res. 39(Database):D612–D619. 20974635 10.1093/nar/gkq1006 PMC3013638

[evaa129-B8] Aurrecoechea C, et al 2017. EuPathDB: the eukaryotic pathogen genomics database resource. Nucleic Acids Res. 45(D1):D581–D591. 27903906 10.1093/nar/gkw1105 PMC5210576

[evaa129-B9] Baumgart E Fahimi HD Steininger H Grabenbauer M. 2003. A review of morphological techniques for detection of peroxisomal (and mitochondrial) proteins and their corresponding mRNAs during ontogenesis in mice: application to the PEX5-knockout mouse with Zellweger syndrome. Microsc Res Tech. 61(2):121–138. 12740819 10.1002/jemt.10322

[evaa129-B10] Beard ME Novikoff AB. 1969. Distribution of peroxisomes (microbodies) in the nephron of the rat. J Cell Biol. 42(2):501–518. 5792337 10.1083/jcb.42.2.501 PMC2107674

[evaa129-B11] Birney E Clamp M Durbin R. 2004. GeneWise and Genomewise. Genome Res. 14(5):988–995. 15123596 10.1101/gr.1865504 PMC479130

[evaa129-B12] Carter RF. 1972. Primary amoebic meningo-encephalitis. An appraisal of present knowledge. Trans R Soc Trop Med Hyg. 66(2):193–213. 4558822 10.1016/0035-9203(72)90147-2

[evaa129-B13] Chelkha N, et al 2018. A phylogenomic study of *Acanthamoeba polyphaga* draft genome sequences suggests genetic exchanges with giant viruses. Front Microbiol. 9:2098. 30237791 10.3389/fmicb.2018.02098 PMC6135880

[evaa129-B14] Cheung N Nagra P Hammersmith K. 2016. Emerging trends in contact lens-related infections. Curr Opin Ophthalmol. 27(4):327–332. 27176217 10.1097/ICU.0000000000000280

[evaa129-B15] Childs GE. 1973a. Diaminobenzidine reactivity of peroxisomes and mitochondria in a parasitic ameba, *Hartmannella culbertsoni*. J Histochem Cytochem. 21(1):26–33. 4694537 10.1177/21.1.26

[evaa129-B16] Childs GE. 1973b. *Hartmannella culbertsoni*: enzymatic, ultrastructural, and cytochemical characteristics of peroxisomes in a density gradient. Exp Parasitol. 34(1):44–55. 4722484 10.1016/0014-4894(73)90061-1

[evaa129-B17] Clarke M, et al 2013. Genome of *Acanthamoeba castellanii* highlights extensive lateral gene transfer and early evolution of tyrosine kinase signaling. Genome Biol. 14(2):R11. 23375108 10.1186/gb-2013-14-2-r11 PMC4053784

[evaa129-B18] Criscuolo A Gribaldo S. 2010. BMGE (Block Mapping and Gathering with Entropy): a new software for selection of phylogenetic informative regions from multiple sequence alignments. BMC Evol Biol. 10(1):210. 20626897 10.1186/1471-2148-10-210 PMC3017758

[evaa129-B19] da Rocha-Azevedo B Costa e Silva-Filho F. 2007. Biological characterization of a clinical and an environmental isolate of *Acanthamoeba polyphaga*: analysis of relevant parameters to decode pathogenicity. Arch Microbiol. 188(5):441–449. 17569030 10.1007/s00203-007-0264-3

[evaa129-B20] De Duve C Baudhuin P. 1966. Peroxisomes (microbodies and related particles). Physiol Rev. 46(2):323–357. 5325972 10.1152/physrev.1966.46.2.323

[evaa129-B21] De Jonckheere JF. 2014. What do we know by now about the genus *Naegleria*? Exp Parasitol. 145:S2–S9. 25108159 10.1016/j.exppara.2014.07.011

[evaa129-B22] Detering H, et al 2015. First draft genome sequence of *Balamuthia mandrillaris*, the causative agent of amoebic encephalitis. Genome Announc. 3(5):e01013–15. 26404594 10.1128/genomeA.01013-15 PMC4582570

[evaa129-B23] Dini LA Cockinos C Frean JA Niszl IA Marku MB. 2000. Unusual case of *Acanthamoeba polyphaga* and *Pseudomonas aeruginosa* keratitis in a contact lens wearer from Gauteng, South Africa. J Clin Microbiol. 38(2):826–829. 10655392 10.1128/jcm.38.2.826-829.2000 PMC86214

[evaa129-B24] Distel B, et al 1996. A unified nomenclature for peroxisome biogenesis factors. J Cell Biol. 135(1):1–3. 8858157 10.1083/jcb.135.1.1 PMC2121017

[evaa129-B25] Do CB Mahabhashyam MSP Brudno M Batzoglou S. 2005. ProbCons: probabilistic consistency-based multiple sequence alignment. Genome Res. 15(2):330–340. 15687296 10.1101/gr.2821705 PMC546535

[evaa129-B26] Fahimi HD Baumgart E. 1999. Current cytochemical techniques for the investigation of peroxisomes: a review. J Histochem Cytochem. 47(10):1219–1232. 10490450 10.1177/002215549904701001

[evaa129-B27] Ferreyra RG, et al 2006. A yeast sterol carrier protein with fatty-acid and fatty-acyl-CoA binding activity. Arch Biochem Biophys. 453(2):197–206. 16890184 10.1016/j.abb.2006.06.024

[evaa129-B28] Fritz-Laylin LK, et al 2010. The genome of *Naegleria gruberi* illuminates early eukaryotic versatility. Cell 140(5):631–642. 20211133 10.1016/j.cell.2010.01.032

[evaa129-B29] Gabaldón T. 2010. Peroxisome diversity and evolution. Philos Trans R Soc Lond B Biol Sci. 365(1541):765–773. 20124343 10.1098/rstb.2009.0240 PMC2817229

[evaa129-B30] Gabaldón T. 2018. Evolution of the peroxisomal proteome. In: del Río LA Schrader M, editors. *Proteomics of peroxisomes: identifying novel functions and regulatory networks. Subcellular Biochemistry.* Vol. 89. Singapore: Springer. p. 221–233. 10.1007/978-981-13-2233-4_9 30378025

[evaa129-B31] Gabaldón T Ginger ML Michels PA. 2016. Peroxisomes in parasitic protists. Mol Biochem Parasitol. 209(1–2):35–45. 26896770 10.1016/j.molbiopara.2016.02.005

[evaa129-B32] Galland N Michels PA. 2010. Comparison of the peroxisomal matrix protein import system of different organisms. Exploration of possibilities for developing inhibitors of the import system of trypanosomatids for anti-parasite chemotherapy. Eur J Cell Biol. 89(9):621–637. 20435370 10.1016/j.ejcb.2010.04.001

[evaa129-B33] Girzalsky W Saffian D Erdmann R. 2010. Peroxisomal protein translocation. Biochim Biophys Acta Mol Cell Res. 1803(6):724–731. 10.1016/j.bbamcr.2010.01.002 20079383

[evaa129-B34] González NH, et al 2011. A single peroxisomal targeting signal mediates matrix protein import in diatoms. PLoS One. 6(9):e25316. 21966495 10.1371/journal.pone.0025316 PMC3178647

[evaa129-B35] González-Robles A Salazar-Villatoro L Omaña-Molina M Lorenzo-Morales J Martínez-Palomo A. 2013. *Acanthamoeba royreba*: morphological features and *in vitro* cytopathic effect. Exp Parasitol. 133(4):369–375. 23357648 10.1016/j.exppara.2013.01.011

[evaa129-B36] González-Robles A, et al 2014. Morphological features and *in vitro* cytopathic effect of *Acanthamoeba griffini* trophozoites isolated from a clinical case. J Parasitol Res. 2014:1–10. 10.1155/2014/256310 PMC4173000 25313337

[evaa129-B37] Greninger AL, et al 2015. Clinical metagenomic identification of *Balamuthia mandrillaris* encephalitis and assembly of the draft genome: the continuing case for reference genome sequencing. Genome Med. 7(1):113. 26620704 10.1186/s13073-015-0235-2 PMC4665321

[evaa129-B38] Guigó R Agarwal P Abril JF Burset M Fickett JW. 2000. An assessment of gene prediction accuracy in large DNA sequences. Genome Res. 10(10):1631–1642. 11042160 10.1101/gr.122800 PMC310940

[evaa129-B39] Hawkins J Bodén M. 2006. Detecting and sorting targeting peptides with neural networks and support vector machines. J Bioinform Comput Biol. 4(1):1–18. 16568539 10.1142/s0219720006001771

[evaa129-B40] Hayashi H Suga T. 1978. Some characteristics of peroxisomes in the slime mould, *Dictyostelium discoideum*. J Biochem. 84(3):513–520. 721792 10.1093/oxfordjournals.jbchem.a132155

[evaa129-B41] Islinger M Cardoso MJR Schrader M. 2010. Be different—the diversity of peroxisomes in the animal kingdom. Biochim Biophys Acta 1803(8):881–897. 20347886 10.1016/j.bbamcr.2010.03.013

[evaa129-B42] Islinger M Grille S Fahimi HD Schrader M. 2012. The peroxisome: an update on mysteries. Histochem Cell Biol. 137(5):547–574. 22415027 10.1007/s00418-012-0941-4

[evaa129-B43] Islinger M Voelkl A Fahimi HD Schrader M. 2018. The peroxisome: an update on mysteries 2.0. Histochem Cell Biol. 150(5):443–471. 30219925 10.1007/s00418-018-1722-5 PMC6182659

[evaa129-B44] Jones P, et al 2014. InterProScan 5: genome-scale protein function classification. Bioinformatics 30(9):1236–1240. 24451626 10.1093/bioinformatics/btu031 PMC3998142

[evaa129-B45] Kalel VC Erdmann R. 2018. Unraveling of the structure and function of peroxisomal protein import machineries. In: del Río LA Schrader M, editors. *Proteomics of peroxisomes: identifying novel functions and regulatory networks. Subcellular Biochemistry*. Vol. 89. Singapore: Springer. p. 299–321. 10.1007/978-981-13-2233-4_13 30378029

[evaa129-B46] Karlyshev AV. 2019. Remarkable features of mitochondrial DNA of *Acanthamoeba polyphaga* Linc Ap-1, revealed by whole-genome sequencing. Microbiol Resour Announc. 8(25):e00430–19. 31221647 10.1128/MRA.00430-19 PMC6588368

[evaa129-B47] Kiel JA Veenhuis M van der Klei IJ. 2006. PEX genes in fungal genomes: common, rare or redundant. Traffic 7(10):1291–1303. 16978390 10.1111/j.1600-0854.2006.00479.x

[evaa129-B48] Kim PK Hettema EH. 2015. Multiple pathways for protein transport to peroxisomes. J Mol Biol. 427(6):1176–1190. 25681696 10.1016/j.jmb.2015.02.005 PMC4726662

[evaa129-B49] Krogh A Larsson B von Heijne G Sonnhammer EL. 2001. Predicting transmembrane protein topology with a hidden Markov model: application to complete genomes. J Mol Biol. 305(3):567–580. 11152613 10.1006/jmbi.2000.4315

[evaa129-B50] Lares-Jiménez LF Booton GC Lares-Villa F Velázquez-Contreras CA Fuerst PA. 2014. Genetic analysis among environmental strains of *Balamuthia mandrillaris* recovered from an artificial lagoon and from soil in Sonora, Mexico. Exp Parasitol. 145:S57–S61. 25076486 10.1016/j.exppara.2014.07.007

[evaa129-B51] Le SQ Gascuel O. 2008. An improved general amino acid replacement matrix. Mol Biol Evol. 25(7):1307–1320. 18367465 10.1093/molbev/msn067

[evaa129-B52] Ledee DR Hay J Byers TJ Seal DV Kirkness CM. 1996. *Acanthamoeba griffini*. Molecular characterization of a new corneal pathogen. Invest Ophthalmol Vis Sci. 37(4):544–550. 8595954

[evaa129-B53] Lemoine F, et al 2019. NGPhylogeny.fr: new generation phylogenetic services for non-specialists. Nucleic Acids Res. 47(W1):W260–W265. 31028399 10.1093/nar/gkz303 PMC6602494

[evaa129-B54] Letunic I Bork P. 2019. Interactive Tree Of Life (iTOL) v4: recent updates and new developments. Nucleic Acids Res. 47(W1):W256–W259. 30931475 10.1093/nar/gkz239 PMC6602468

[evaa129-B55] Liechti N Schürch N Bruggmann R Wittwer M. 2018. The genome of *Naegleria lovaniensis*, the basis for a comparative approach to unravel pathogenicity factors of the human pathogenic amoeba *N. fowleri*. BMC Genomics. 19(1):654. 30185166 10.1186/s12864-018-4994-1 PMC6125883

[evaa129-B56] Lingard MJ Trelease RN. 2006. Five *Arabidopsis* peroxin 11 homologs individually promote peroxisome elongation, duplication or aggregation. J Cell Sci. 119(9):1961–1972. 16636080 10.1242/jcs.02904

[evaa129-B57] Ludewig-Klingner AK Michael V Jarek M Brinkmann H Petersen J. 2018. Distribution and evolution of peroxisomes in Alveolates (Apicomplexa, Dinoflagellates, Ciliates). Genome Biol Evol. 10(1):1–13. 29202176 10.1093/gbe/evx250 PMC5755239

[evaa129-B58] Makiuchi T Nozaki T. 2014. Highly divergent mitochondrion-related organelles in anaerobic parasitic protozoa. Biochimie 100:3–17. 24316280 10.1016/j.biochi.2013.11.018

[evaa129-B59] Marchler-Bauer A Bryant SH. 2004. CD-Search: protein domain annotations on the fly. Nucleic Acids Res. 32(Web Server):W327–W331. 15215404 10.1093/nar/gkh454 PMC441592

[evaa129-B60] Marciano-Cabral F Puffenbarger R Cabral GA. 2000. The increasing importance of *Acanthamoeba* infections. J Eukaryot Microbiol. 47(1):29–36. 10651293 10.1111/j.1550-7408.2000.tb00007.x

[evaa129-B61] Martínez AJ. 1991. Infection of the central nervous system due to *Acanthamoeba*. Rev Infect Dis. 13:S399–S402. 2047674 10.1093/clind/13.supplement_5.s399

[evaa129-B62] Matsumoto N Tamura S Fujiki Y. 2003. The pathogenic peroxin Pex26p recruits the Pex1p–Pex6p AAA ATPase complexes to peroxisomes. Nat Cell Biol. 5(5):454–460. 12717447 10.1038/ncb982

[evaa129-B63] Matsunaga S, et al 1998. Chromosome size polymorphisms in the genus *Acanthamoeba*: electrokaryotype by pulsed-field gel electrophoresis. Protist 149(4):323–340. 23194715 10.1016/S1434-4610(98)70039-2

[evaa129-B64] Miller MA Pfeiffer W Schwartz T. 2010. Creating the CIPRES Science Gateway for inference of large phylogenetic trees. In: Proceedings of the Gateway Computing Environments Workshop (GCE); New Orleans, LA. p. 1–8.

[evaa129-B65] Moog D Przyborski JM Maier UG. 2017. Genomic and proteomic evidence for the presence of a peroxisome in the Apicomplexan parasite *Toxoplasma gondii* and other Coccidia. Genome Biol Evol. 9(11):3108–3121. 29126146 10.1093/gbe/evx231 PMC5737649

[evaa129-B66] Müller M. 1975. Biochemistry of protozoan microbodies: peroxisomes, alpha-glycerophosphate oxidase bodies, hydrogenosomes. Annu Rev Microbiol. 29:467–483. 1180522 10.1146/annurev.mi.29.100175.002343

[evaa129-B67] Müller M MØller KM. 1969. Urate oxidase and its association with peroxisomes in *Acanthamoeba* sp. Eur J Biochem. 9(3):424–430. 5795517 10.1111/j.1432-1033.1969.tb00626.x

[evaa129-B68] Needleman SB Wunsch CD. 1970. A general method applicable to the search for similarities in the amino acid sequence of two proteins. J Mol Biol. 48(3):443–453. 5420325 10.1016/0022-2836(70)90057-4

[evaa129-B69] Neuberger G Maurer-Stroh S Eisenhaber B Hartig A Eisenhaber F. 2003. Prediction of peroxisomal targeting signal 1 containing proteins from amino acid sequence. J Mol Biol. 328(3):581–592. 12706718 10.1016/s0022-2836(03)00319-x

[evaa129-B70] Niederkorn JY Alizadeh H Leher H McCulley JP. 1999. The pathogenesis of *Acanthamoeba* keratitis. Microbes Infect. 1(6):437–443. 10602676 10.1016/s1286-4579(99)80047-1

[evaa129-B71] Omaña-Molina M, et al 2016. *Acanthamoeba* genotypes T3 and T4 as causative agents of amoebic keratitis in Mexico. Parasitol Res. 115(2):873–878. 26581373 10.1007/s00436-015-4821-4

[evaa129-B72] Opperdoes FR Butenko A Flegontov P Yurchenko V Lukeš J. 2016. Comparative metabolism of free-living *Bodo saltans* and parasitic trypanosomatids. J Eukaryot Microbiol. 63(5):657–678. 27009761 10.1111/jeu.12315

[evaa129-B73] Opperdoes FR De Jonckheere JF Tielens AG. 2011. *Naegleria gruberi* metabolism. Int J Parasitol. 41(9):915–924. 21722646 10.1016/j.ijpara.2011.04.004

[evaa129-B74] Padhi TR, et al 2017. Ocular parasitoses: a comprehensive review. Surv Ophthalmol. 62(2):161–189. 27720858 10.1016/j.survophthal.2016.09.005

[evaa129-B75] Paight C Slamovits CH Saffo MB Lane CE. 2019. *Nephromyces* encodes a urate metabolism pathway and predicted peroxisomes, demonstrating that these are not ancient losses of Apicomplexans. Genome Biol Evol. 11(1):41–53. 30500900 10.1093/gbe/evy251 PMC6320678

[evaa129-B76] Parish RW. 1975. Mitochondria and peroxisomes from the cellular slime mould *Dictyostelium discoideum*. Isolation techniques and urate oxidase association with peroxisomes. Eur J Biochem. 58(2):523–531. 241646 10.1111/j.1432-1033.1975.tb02401.x

[evaa129-B77] Pieuchot L Jedd G. 2012. Peroxisome assembly and functional diversity in eukaryotic microorganisms. Annu Rev Microbiol. 66(1):237–263. 22994494 10.1146/annurev-micro-092611-150126

[evaa129-B78] Platta HW Erdmann R. 2007. Peroxisomal dynamics. Trends Cell Biol. 17(10):474–484. 17913497 10.1016/j.tcb.2007.06.009

[evaa129-B79] Platta HW, et al 2016. Regulation of peroxisomal matrix protein import by ubiquitination. Biochim Biophys Acta 1863(5):838–849. 26367801 10.1016/j.bbamcr.2015.09.010

[evaa129-B80] Reyes-Batlle M, et al 2014. Isolation and characterization of *Acanthamoeba* strains from soil samples in Gran Canaria, Canary Islands, Spain. Parasitol Res. 113(4):1383–1388. 24449449 10.1007/s00436-014-3778-z

[evaa129-B81] Salvatore M Shu N Elofsson A. 2018. The SubCons webserver: a user friendly web interface for state-of-the-art subcellular localization prediction. Protein Sci. 27(1):195–201. 28901589 10.1002/pro.3297 PMC5734273

[evaa129-B82] Sayers EW, et al 2019. GenBank. Nucleic Acids Res. 47(D1):D94–D99. 30365038 10.1093/nar/gky989 PMC6323954

[evaa129-B83] Schlüter A, et al 2006. The evolutionary origin of peroxisomes: an ER-peroxisome connection. Mol Biol Evol. 23(4):838–845. 16452116 10.1093/molbev/msj103

[evaa129-B84] Schrader M Bonekamp NA Islinger M. 2012. Fission and proliferation of peroxisomes. Biochim Biophys Acta Mol Basis Dis. 1822(9):1343–1357. 10.1016/j.bbadis.2011.12.014 22240198

[evaa129-B85] Schrader M Fahimi HD. 2008. The peroxisome: still a mysterious organelle. Histochem Cell Biol. 129(4):421–440. 18274771 10.1007/s00418-008-0396-9 PMC2668598

[evaa129-B86] Schuster FL Visvesvara GS. 1996. Axenic growth and drug sensitivity studies of *Balamuthia mandrillaris*, an agent of amebic meningoencephalitis in humans and other animals. J Clin Microbiol. 34(2):385–388. 8789020 10.1128/jcm.34.2.385-388.1996 PMC228802

[evaa129-B87] Sharma S Pasricha G Das D Aggarwal RK. 2004. *Acanthamoeba* keratitis in non-contact lens wearers in India: DNA typing-based validation and a simple detection assay. Arch Ophthalmol. 122(10):1430–1434. 15477452 10.1001/archopht.122.10.1430

[evaa129-B88] Sibirny AA. 2016. Yeast peroxisomes: structure, functions and biotechnological opportunities. FEMS Yeast Res. 16(4):fow038. 27189367 10.1093/femsyr/fow038

[evaa129-B89] Smith JJ Aitchison JD. 2013. Peroxisomes take shape. Nat Rev Mol Cell Biol. 14(12):803–817. 24263361 10.1038/nrm3700 PMC4060825

[evaa129-B90] Stamatakis A. 2014. RAxML version 8: a tool for phylogenetic analysis and post-analysis of large phylogenies. Bioinformatics 30(9):1312–1313. 24451623 10.1093/bioinformatics/btu033 PMC3998144

[evaa129-B91] Thoms S Erdmann R. 2005. Dynamin-related proteins and Pex11 proteins in peroxisome division and proliferation. FEBS J. 272(20):5169–5181. 16218949 10.1111/j.1742-4658.2005.04939.x

[evaa129-B92] Tsirigos KD Peters C Shu N Käll L Elofsson A. 2015. The TOPCONS web server for consensus prediction of membrane protein topology and signal peptides. Nucleic Acids Res. 43(W1):W401–W407. 25969446 10.1093/nar/gkv485 PMC4489233

[evaa129-B93] UniProt Consortium. 2019. UniProt: a worldwide hub of protein knowledge. Nucleic Acids Res. 47(D1):D506–D515. 30395287 10.1093/nar/gky1049 PMC6323992

[evaa129-B94] Visvesvara GS. 2013. Infections with free-living amebae. In: García HH Tanowitz HB del Brutto OH, editors. *Neuroparasitology and Tropical Neurology. Handbook of Clinical Neurology*. Vol. 114. Amsterdam: Elsevier. p. 153–168. 10.1016/B978-0-444-53490-3.00010-8 23829906

[evaa129-B95] Visvesvara GS Moura H Schuster FL. 2007. Pathogenic and opportunistic free-living amoebae: *Acanthamoeba* spp., *Balamuthia mandrillaris*, *Naegleria fowleri*, and *Sappinia diploidea*. FEMS Immunol Med Microbiol. 50(1):1–26. 17428307 10.1111/j.1574-695X.2007.00232.x

[evaa129-B96] Visvesvara GS Schuster FL Martinez AJ. 1993. *Balamuthia mandrillaris*, N. G., N. Sp., agent of amebic meningoencephalitis in humans and other animals. J Eukaryot Microbiol. 40(4):504–514. 8330028 10.1111/j.1550-7408.1993.tb04943.x

[evaa129-B97] Wanders RJA Waterham HR. 2006. Biochemistry of mammalian peroxisomes revisited. Annu Rev Biochem. 75(1):295–332. 16756494 10.1146/annurev.biochem.74.082803.133329

[evaa129-B98] Waterhouse RM, et al 2018. BUSCO applications from quality assessments to gene prediction and phylogenomics. Mol Biol Evol. 35(3):543–548. 29220515 10.1093/molbev/msx319 PMC5850278

[evaa129-B99] Yeh RF Lim LP Burge CB. 2001. Computational inference of homologous gene structures in the human genome. Genome Res. 11(5):803–816. 11337476 10.1101/gr.175701 PMC311055

[evaa129-B100] Yu CS, et al 2014. CELLO2GO: a web server for protein subCELlular LOcalization prediction with functional Gene Ontology annotation. PLoS One 9(6):e99368. 24911789 10.1371/journal.pone.0099368 PMC4049835

[evaa129-B101] Žárský V Tachezy J. 2015. Evolutionary loss of peroxisomes—not limited to parasites. Biol Direct 10(1):74. 26700421 10.1186/s13062-015-0101-6 PMC4690255

[evaa129-B102] Zysset-Burri DC, et al 2014. Genome-wide identification of pathogenicity factors of the free-living amoeba *Naegleria fowleri*. BMC Genomics. 15(1):496. 24950717 10.1186/1471-2164-15-496 PMC4082629

