# Erratum to: Ultrastructural, Cytochemical, and Comparative Genomic Evidence of Peroxisomes in Three Genera of Pathogenic Free-Living Amoebae, Including the First Morphological Data for the Presence of This Organelle in Heteroloboseans

**DOI:** 10.1093/gbe/evab180

**Published:** 2021-08-21

**Authors:** Arturo González-Robles, Mónica González-Lázaro, Anel Edith Lagunes-Guillén, Maritza Omaña-Molina, Luis Fernando Lares-Jiménez, Fernando Lares-Villa, Adolfo Martínez-Palomo

*Genome Biology and Evolution*, Volume 12, Issue 10, October 2020, Pages 1734–1750, https://doi.org/10.1093/gbe/evaa129

In the originally published version of this manuscript, the supplementary data were incomplete and have been amended to include another 11 files which were missing. This error has been corrected online. The publisher apologizes for the error.

