# Supplementary material for: Ultrastructural, Cytochemical, and Comparative Genomic Evidence of Peroxisomes in Three Genera of Pathogenic Free-Living Amoebae, Including the First Morphological Data for the Presence of This Organelle in Heteroloboseans
Source: Genome Biol Evol. 2020 Jun 30;12(10):1734–50. doi: 10.1093/gbe/evaa129 (PMC7549135; doi:10.1093/gbe/evaa129)
Supplement: evaa129_Supplementary_Data [file evaa129_supplementary_data.zip › evaa129-suppl_data/Supplementary files legends.pdf]

Supplementary Files Legends

Supplementary table 1. Accession numbers of various peroxisomal proteins from *Homo sapiens*, *Naegleria gruberi* and *Dictyostelium discoideum*. Accession numbers correspond to entries in the UniProt database. These sequences were used as queries for BLASTp or tBLASTn analyses to identify putative peroxin and catalase homologues in *A. castellanii*, *B. mandrillaris*, *N. fowleri* and *N. lovaniensis*.

Supplementary table 2. Predicted peroxin and catalase protein sequences identified in *Acanthamoeba castellanii*. Accession numbers correspond to entries in AmoebaDB, release 46. The top *E*-values from BLASTp analyses using the protein sequences of peroxins from *Homo sapiens* and *Naegleria gruberi* as queries are shown. Results of reciprocal BLASTp analyses against the query databases are also indicated. Values marked with an asterisk correspond to BLASTp analyses in which the length of the seed that initiates the alignment (word size) was changed from 6 (default value) to 3. To confirm the identity of the putative homologues, their conserved domains were identified using NCBI's Conserved Domain Search (CDS). Peroxins functions were compiled from Platta and Erdmann (2007); Žárský and Tachezy (2015) and Platta et al. (2016).

Abbreviations: Catalase-rel: catalase-related immune responsive family (small conserved region within catalase enzymes); CDC48: AAA family ATPase, CDC48 subfamily; katE: hydroperoxidase II (catalase); PHD: plant homeodomain finger motif (this motif resembles the metal binding RING domain); PMP: peroxisomal membrane protein; PTS: peroxisomal targeting sequence; RING: really interesting new gene domain; RING-HC: RING finger protein, HC subclass; mRING: modified RING finger protein; SAD_SRA: SET and RING

finger Associated, YDG motif; SH3: Src homology 3 domain; TPR: tetratricopeptide repeat; UQ_con: ubiquitin-conjugating enzyme; WD40: 40 amino acid long motif, often terminating in a Trp-Asp (W-D) dipeptide.

[a] Although the BLASTp *E*-value of these sequences was above the cutoff (1e-04), they were considered as putative homologues since the NCBI CDS algorithm identified the presence of the relevant peroxin domains.

[b] This domain was identified using InterProScan 5.

[#] Predicted peroxisomal targeting sequence (PTS1).

Supplementary tables 3 and 4. Predicted peroxin and catalase protein sequences identified in *Acanthamoeba polyphaga* (supplementary table 3) and *Acanthamoeba royreba* (supplementary table 4). Accession numbers and scaffold names correspond to entries in the *A. polyphaga* or *A. royreba* NCBI Whole Genome Shotgun (WGS) databases. The coordinates on the scaffold where each putative peroxin gene is predicted to be located are also indicated. A double asterisk indicates the coordinates on the reverse complement sequence of the scaffold. The top *E*-values from tBLASTn analyses using the protein sequences of different peroxins from *A. castellanii* as queries are shown. To confirm the identity of the putative homologues, the conserved domains were identified using NCBI's CDS. Results of reciprocal BLASTp analyses against the query database are also indicated.

Abbreviations: UBCc: Ubiquitin-conjugating enzyme E2, catalytic domain.

[a] This domain was identified using InterProScan 5.

[b] A longer version of this protein was identified using the GENSCAN software (http://hollywood.mit.edu/GENSCAN.html; last accessed May 10, 2020) (Burge and Karlin, 1997). This putative protein sequence is 173 amino acids long and it is predicted to contain the PEX22 domain:

```
>GENSCAN_predicted_peptide_5|173_aa
MANLSWWCRWLVGARTARVVAVAVVLVAGGGLVAVLARLRLRGRLAPPPPEDHLITVPQV
VLAVNMTTGLIDLVVEALPVLAELASRADLYLITQCEDDRAEQRVRDLLAGCGIYEAGLN
PHSLTVGYPLTFGQKVLFCGTSVGRVHMARQLEVFMHIDGTTAWGAPLRVAQY
```

§ Sequences identified using *A. castellanii* Pex11 ACA1_053960 as query.

‡ Sequences identified using *A. castellanii* Pex11 ACA1_297780 as query.

¶ Sequences identified using *A. castellanii* Pex11 ACA1_279800 as query.

# Predicted peroxisomal targeting sequence (PTS1).

Supplementary table 5. Predicted peroxin and catalase protein sequences identified in *Balamuthia mandrillaris*. Accession numbers and scaffold names correspond to entries in the *B. mandrillaris* NCBI WGS database. The coordinates on the scaffold where each putative peroxin gene is predicted to be located are also indicated. A double asterisk indicates the coordinates on the reverse complement sequence of the scaffold. The top *E*-values from tBLASTn analyses using the protein sequences of different peroxins from *H. sapiens*, *N. gruberi* and *A. castellanii* as queries are shown. The percentage of query cover and of sequence identity are also indicated. To confirm the identity of the putative homologues, their conserved domains were identified using NCBI's CDS. Results of

reciprocal BLASTp analyses against the query databases (*H. sapiens*, *N. gruberi* and *A. castellanii*) are also indicated. Values marked with an asterisk correspond to BLASTp analyses in which the length of the seed that initiates the alignment (word size) was changed from 6 (default value) to 3.

Abbreviations: COG5078: ubiquitin-protein ligase; SpoVK: AAA+-type ATPase, SpoVK/Ycf46/Vps4 family.

[a] Although the tBLASTn and the reciprocal BLASTp *E*-values of these sequences are above the cutoff (1e-04), they were considered as putative homologues since the NCBI CDS algorithm identified the presence of the functional domains and because the BLAST *E*-values obtained with the *A. castellanii* queries were below the cutoff.

[b] Although this Pex19 sequence had a tBLASTn *E*-value above the cutoff and the NCBI CDS algorithm could not identify a conserved domain (likely because the sequence is only 54 amino acids long), it was considered as a putative homologue since it is a perfect match to the C-terminal region of the Pex19 sequence found in the LFUI01000013.1 scaffold.

[c] A longer version of this protein was identified using the GENSCAN software (http://hollywood.mit.edu/GENSCAN.html; last accessed May 10, 2020) (Burge and Karlin, 1997). This putative protein sequence is 484 amino acids long and it is predicted to contain the PEX14 domain:

```
>GENSCAN_predicted_peptide_98|484_aa
MLAEPQEEAQAAEGAPPASEPNPSSVSATEAEADAEDERKRELAKRKEELAKRQQQARVK
RGGSPPSSAASGTKGKEKADEEATPASTPAPAPKPKVIREEQVQNALKFLTHPKVQSSPL
GKRIAFLEHKGLNDDEISEALKRANVSSAEIKAAAAATAATTSVAAPVSSSSLQSSVPLP
SQQQAPSKRDNNGAGNGALLEAAAERKAEAEEAEKERRMKQELDAFRSSQTSLNNEMKQA
FLEMKQMFEQSRSTNTQPSQPSSSSPLSPSSSPSTTSQTPNENSVVLSREELIALKSELK
ALKAMVHDQRMAAATIPIPENPISKTRSTPSSGGGLSSSLPSWMMERRTSAPTASSGGVE
EVEKKEEGKDEDKGKQIEQKEEVEQNKTVSPSPLKNAITKKPYQRKKEALLQQKQEALAP
PPTTTTDVASPVSPSSERKYSFPVSSASVAVPWPSSTASSTTTTEASSPASSDSTPQQQE
QQAE
```

[d] This domain was identified using InterProScan 5.

[§] Sequence identified using *A. castellanii* Pex11 ACA1_053960 as query.

[‡] Sequences identified using *A. castellanii* Pex11 ACA1_297780 as query.

[#] Predicted peroxisomal targeting sequence (PTS1).

Supplementary table 6. Predicted peroxin and catalase protein sequences identified in *Naegleria fowleri*. Accession numbers correspond to entries in AmoebaDB, release 46. The top *E*-values from BLASTp analyses using the protein sequences of peroxins from *Homo sapiens* and *Naegleria gruberi* as queries are shown. Results of reciprocal BLASTp analyses against the query databases are also indicated. Values marked with an asterisk correspond to BLASTp analyses in which the length of the seed that initiates the alignment (word size) was changed from 6 (default value) to 3. To confirm the identity of the putative homologues, their conserved domains were identified using NCBI's CDS.

[a] Although the BLASTp *E*-value of these sequences was above the cutoff (1e-04), they were considered as putative homologues since the NCBI CDS algorithm identified the presence of the relevant peroxin domains.

[b] A longer version of this protein, which includes both the PEX2_PEX12 and the RING-HC_PEX2 domains, was identified using the Pex2 sequence from *D. discoideum* as query for a tBLASTn analysis against the *N. fowleri* Whole Genome Shotgun database stored in NCBI (*N. fowleri* WGS ID: AWXF01000367.1; scaffold name: *N. fowleri* strain ATCC 30863 Nfow_contig_367, WGSS; coordinates on scaffold (NCBI): 463 - 912 (+); coordinates on scaffold (GeneWise): 214 - 1585 (+); tBLASTn *E*-value: 1e-15, query cover: 73%, identity: 33%). Results of a reciprocal BLASTp analysis against the *D. discoideum* protein database confirmed the homology between the query and the newly identified *N. fowleri* sequence (BLASTp *E*-value: 2e-48, query cover: 78%, identity: 34%).

[c] This domain was identified using InterProScan 5.

[#] Predicted peroxisomal targeting sequence (PTS1).

Supplementary table 7. Predicted peroxin and catalase protein sequences identified in *Naegleria lovaniensis*. Accession numbers and scaffold names correspond to entries in the *N. lovaniensis* NCBI WGS database. The coordinates on the scaffold where each putative peroxin gene is predicted to be located are also indicated. A double asterisk indicates the coordinates on the reverse complement sequence of the scaffold. The top *E*-values from tBLASTn analyses using the protein sequences of different peroxins from *D. discoideum*, *N. gruberi* and *N. fowleri* as queries are shown. The percentage of query

cover and of sequence identity are also indicated. To confirm the identity of the putative homologues, their conserved domains were identified using NCBI's CDS. Results of reciprocal BLASTp analyses against the query databases (*D. discoideum*, *N. gruberi* and *N. fowleri*) are also shown, including the percentage of query cover and of sequence identity. For *N. fowleri*, these percentages are not available, as these values are not provided by the AmoebaDB BLAST tool and NCBI does not have a complete protein database for this amoeba.

[a] This domain was identified using InterProScan.

[§] Sequence identified using *N. fowleri* Pex11 NF0113850 as query.

[‡] Sequence identified using *N. fowleri* Pex11 NF0009070 as query.

[#] Predicted peroxisomal targeting sequence (PTS1).

Supplementary table 8. Predicted peroxin protein sequences identified in *Naegleria gruberi*. Accession numbers and scaffold names correspond to entries in the *N. gruberi* NCBI WGS database (https://www.ncbi.nlm.nih.gov/nuccore/ACER00000000.1; last accessed February 1, 2020). The coordinates on the scaffold where each putative peroxin gene is predicted to be located are also indicated. A double asterisk indicates the coordinates on the reverse complement sequence of the scaffold. The top *E*-values from tBLASTn analyses using the protein sequences from *N. fowleri* as queries are shown. The percentage of query cover and of sequence identity are also indicated. To confirm the identity of the putative homologues, their conserved domains were identified using NCBI's

CDS. Results of reciprocal BLASTp analyses against the query database are also indicated.

§ Sequence identified using *N. fowleri* Pex11 NF0113850 as query. This sequence is available in the UniProt database under accession number D2V0G7.

‡ Sequence identified using *N. fowleri* Pex11 NF0009070 as query.

Supplementary table 9. Identification of PTS1 in the predicted catalase sequences identified in three genera of pathogenic free-living amoebae. Results of targeting prediction analyses performed with CELLO2GO, DeepLoc-1.0, Pprowler 1.2, PTS1 Predictor, Subcons and SignalP-5.0 are shown. The numbers in parenthesis correspond to the targeting score / likelihood. Sequences were also analyzed for the presence of potential transmembrane domains with TOPCONS 2.0 and TMHMM 2.0. Proteins were considered to be targeted to the peroxisome if not more than one prediction differed from the others and if the sequence was not predicted to have transmembrane domains.

Supplementary fig. 1. Analysis of the genome assemblies of various Amoebozoans and Heteroloboseans using Benchmarking Universal Single-copy Orthologs (BUSCOs). The completeness of the different Amoebozoan and Heterolobosean genome assemblies used in this study was assessed by analyzing 303 conserved BUSCOs from the Eukaryota dataset. A high number of duplicated BUSCOs was found in the *A. polyphaga* CDFK assembly, the *A. royreba* CDEZ assembly and in the *B. mandrillaris*

LFUI assembly. The high number of duplicated BUSCOs suggests that haplotypes were not properly collapsed during the genome assembly procedure.

Supplementary text 1. Alignment of the predicted Pex11 protein sequences identified in various Amoebozoans and Heteroloboseans. The amino acid identity between the different members of the Pex11 family found in *A. castellanii*, *A. polyphaga*, *A. royreba* and *B. mandrillaris* (Amoebozoa), as well as those found in *N. gruberi*, *N. fowleri* and *N. lovaniensis* (Heterolobosea) were determined using the EMBOSS Needle software.

Supplementary text 2. Alignment of the predicted *Acanthamoeba polyphaga* Pex12 CDFK01222939.1 and CDFK01184659.1 protein sequences. When the sequence bearing the PEX2_PEX12 domain (CDFK01222939.1) is aligned with the sequence containing the mRING_PEX12 domain (CDFK01184659.1), there is a small area over which they overlap, which suggests that they could be part of the same sequence. These putative Pex12 protein sequences were aligned using the EMBOSS Needle software.

References:

Burge C, Karlin S. 1997. Prediction of complete gene structures in human genomic DNA. J Mol Biol. 268:78-94.
