# Supplementary material for: Ultrastructural, Cytochemical, and Comparative Genomic Evidence of Peroxisomes in Three Genera of Pathogenic Free-Living Amoebae, Including the First Morphological Data for the Presence of This Organelle in Heteroloboseans
Source: Genome Biol Evol. 2020 Jun 30;12(10):1734–50. doi: 10.1093/gbe/evaa129 (PMC7549135; doi:10.1093/gbe/evaa129)
Supplement: evaa129_Supplementary_Data [file evaa129_supplementary_data.zip › evaa129-suppl_data/Supplementary table 9 - Identification of PTS1 in the predicted catalase sequences identified in three genera of pathogenic free-living amoebae.pdf]

Supplementary table 9 - Identification of PTS1 in the predicted catalase sequences identified in three genera of pathogenic free-living amoebae

Protein	Sequence ID	Protein sequence	Protein length (aa)	CELLO2GO	DeepLoc-1.0	PProwler 1.2	PTS1 Predictor	Subcons	SignalP5.0	TMHMM 2.0	TOPCONS	Notes
A. castellanii catalase	ACA1_014370	>ACA1_014370 [illegible]	protein_length=726	Peroxisomal (2.240) Cytoplasmic (1.118)	Cytoplasm (0.6678) soluble (0.9948)	Peroxisome (0.00) Other (0.95)	Not targeted (-96.738)	Peroxisomal	SignalPeptide=No Loc=Other (0.9976)	PredHelix=0	SigPep=No TM-reg=No	
A. castellanii catalase	ACA1_031660	>ACA1_031660 [illegible]	protein_length=487	Peroxisomal (2.357)	Peroxisome (0.7414) soluble (0.9414)	Peroxisome (0.61) Other (0.96)	Targeted (10.249)	Peroxisomal	SignalPeptide=No Loc=Other (0.9973)	PredHelix=0	SigPep=No TM-reg=No	**Most likely peroxisomal**
A. castellanii catalase	ACA1_146420	>ACA1_146420 [illegible]	protein_length=154	Mitochondrial (1.854) Nuclear (1.265)	Mitochondrion (0.4549) soluble (0.8759)	Peroxisome (0.00) Other (0.96)	Not targeted (-64.359)	Mitochondrial	SignalPeptide=No Loc=Other(0.9968)	PredHelix=0	SigPep=No TM-reg=No	
A. castellanii catalase	ACA1_146430	>ACA1_146430 [illegible]	protein_length=537	Peroxisomal (2.149) Mitochondrial (1.000)	Peroxisome (0.5444) soluble (0.8947)	Peroxisome (0.00) Other (0.97)	Not targeted (-64.359)	Peroxisomal	SignalPeptide=No Loc=Other (0.9972)	PredHelix=0	SigPep=No TM-reg=No	
A. polyphaga catalase	CDFK01185444.1	>CDFK01185444.1 [illegible]	protein_length=224	Mitochondrial (2.395) Nuclear (1.258)	Mitochondrion (0.2607) soluble (0.8373)	Peroxisome (0.00) Other (0.97)	Not targeted (-70.387)	Cytoplasmic	SignalPeptide=No Loc=Other (0.9983)	PredHelix=0	SigPep=No TM-reg=No	
A. polyphaga catalase	CDFK01193492.1	>CDFK01193492.1 [illegible]	protein_length=616	Peroxisomal (1.445) Cytoplasmic (1.567)	Cytoplasm (0.6102) soluble (0.995)	Peroxisome (0.00) Other (0.97)	Not targeted (-64.142)	Cytoplasmic	SignalPeptide=No Loc=Other (0.9988)	PredHelix=0	SigPep=No TM-reg=No	
A. polyphaga catalase	CDFK01199395.1	>CDFK01199395.1 [illegible]	protein_length=487	Peroxisomal (2.357)	Peroxisome (0.7415) soluble (0.9415)	Peroxisome (0.42) Other (0.97)	Targeted (10.348)	Peroxisomal	SignalPeptide=No Loc=Other (0.999)	PredHelix=0	SigPep=No TM-reg=No	**Most likely peroxisomal**
A. polyphaga catalase	CDFK01200488.1	>CDFK01200488.1 [illegible]	protein_length=246	Mitochondrial (1.168)	Peroxisome (0.5395) soluble (0.9281)	Peroxisome (0.00) Other (0.96)	Not targeted (-90.802)	Mitochondrial	SignalPeptide=No Loc=Other (0.9971)	PredHelix=0	SigPep=No TM-reg=No	
A. polyphaga catalase	CDFK01202075.1	>CDFK01202075.1 [illegible]	protein_length=726	Peroxisomal (2.391) Cytoplasmic (1.109)	Cytoplasm (0.6604) soluble (0.9929)	Peroxisome (0.00) Other (0.96)	Not targeted (-10.849)	Peroxisomal	SignalPeptide=No Loc=Other (0.998)	PredHelix=0	SigPep=No TM-reg=No	
A. polyphaga catalase	CDFK01205050.1	>CDFK01205050.1 [illegible]	protein_length=537	Peroxisomal (1.741) Mitochondrial (1.070)	Mitochondrion (0.3615) soluble (0.7779)	Peroxisome (0.00) Other (0.96)	Not targeted (-80.021)	Mitochondrial	SignalPeptide=No Loc=Other (0.9966)	PredHelix=0	SigPep=No TM-reg=No	
A. polyphaga catalase	CDFK01206097.1	>CDFK01206097.1 [illegible]	protein_length=473	Peroxisomal (2.488) Cytoplasmic (1.004)	Peroxisome (0.6817) soluble (0.9253)	Peroxisome (0.43) Other (0.96)	Targeted (10.348)	Peroxisomal	SignalPeptide=No Loc=Other (0.9989)	PredHelix=0	SigPep=No TM-reg=No	**Most likely peroxisomal**
A. polyphaga catalase	CDFK01207629.1	>CDFK01207629.1 [illegible]	protein_length=163	Cytoplasmic (2.205) Mitochondrial (1.081)	Cytoplasm (0.4891) soluble (0.9594)	Peroxisome (0.00) Other (0.96)	Not targeted (-46.494)	Cytoplasmic	SignalPeptide=No Loc=Other (0.998)	PredHelix=0	SigPep=No TM-reg=No	
A. polyphaga catalase	CDFK01208008.1	>CDFK01208008.1 [illegible]	protein_length=473	Peroxisomal (2.488) Cytoplasmic (1.004)	Peroxisome (0.6817) soluble (0.9253)	Peroxisome (0.43) Other (0.96)	Targeted (10.348)	Peroxisomal	SignalPeptide=No Loc=Other (0.9989)	PredHelix=0	SigPep=No TM-reg=No	**Most likely peroxisomal**
A. polyphaga catalase	CDFK01208009.1	>CDFK01208009.1 [illegible]	protein_length=487	Peroxisomal (2.357)	Peroxisome (0.7415) soluble (0.9415)	Peroxisome (0.42) Other (0.97)	Targeted (10.348)	Peroxisomal	SignalPeptide=No Loc=Other (0.999)	PredHelix=0	SigPep=No TM-reg=No	**Most likely peroxisomal**
A. polyphaga catalase	CDFK01209054.1	>CDFK01209054.1 [illegible]	protein_length=246	Mitochondrial (1.137)	Peroxisome (0.4847) soluble (0.8968)	Peroxisome (0.00) Other (0.96)	Not targeted (-90.802)	Mitochondrial	SignalPeptide=No Loc=Other (0.9967)	PredHelix=0	SigPep=No TM-reg=No	
A. polyphaga catalase	CDFK01221902.1	>CDFK01221902.1 [illegible]	protein_length=583	Peroxisomal (1.973) Cytoplasmic (1.320)	Mitochondrion (0.3103) soluble (0.8492)	Peroxisome (0.00) Other (0.96)	Not targeted (-28.126)	Peroxisomal	SignalPeptide=No Loc=Other (0.9979)	PredHelix=0	SigPep=No TM-reg=No	
A. polyphaga catalase	CDFK01185398.1	>CDFK01185398.1 [illegible]	protein_length=318	Peroxisomal (1.028) Cytoplasmic (1.243)	Mitochondrion (0.5334) soluble (0.7992)	Peroxisome (0.00) Other (0.96)	Not targeted (-44.974)	Mitochondrial	SignalPeptide=No Loc=Other (0.9965)	PredHelix=0	SigPep=No TM-reg=No	
A. polyphaga catalase	CDFK01205051.1	>CDFK01205051.1 [illegible]	protein_length=317	Cytoplasmic (1.290) Mitochondrial (1.337)	Cytoplasm (0.5452) soluble (0.9506)	Peroxisome (0.00) Other (0.96)	Not targeted (-76.128)	Peroxisomal	SignalPeptide=No Loc=Other (0.9993)	PredHelix=0	SigPep=No TM-reg=No	
A. polyphaga catalase	CDFK01216617.1	>CDFK01216617.1 [illegible]	protein_length=320	Peroxisomal (1.006) Cytoplasmic (1.082) Mitochondrial (1.509)	Cytoplasm (0.6702) soluble (0.976)	Peroxisome (0.00) Other (0.96)	Not targeted (-76.128)	Peroxisomal	SignalPeptide=No Loc=Other (0.9983)	PredHelix=0	SigPep=No TM-reg=No	
A. polyphaga catalase	LQHA01002274.1	>LQHA01002274.1 [illegible]	protein_length=655	Peroxisomal (2.287)	Cytoplasm (0.4131) soluble (0.9517)	Peroxisome (0.00) Other (0.96)	Not targeted (-96.738)	Peroxisomal	SignalPeptide=No Loc=Other (0.9992)	PredHelix=0	SigPep=No TM-reg=No	
A. polyphaga catalase	LQHA01003761.1	>LQHA01003761.1 [illegible]	protein_length=534	Peroxisomal (1.909) Mitochondrial (1.119)	Peroxisome (0.609) soluble (0.9435)	Peroxisome (0.00) Other (0.96)	Not targeted (-76.128)	Mitochondrial	SignalPeptide=No Loc=Other (0.9971)	PredHelix=0	SigPep=No TM-reg=No	
A. polyphaga catalase	LQHA01009034.1	>LQHA01009034.1 [illegible]	protein_length=310	Peroxisomal (1.602)	Mitochondrion (0.6139) soluble (0.719)	Peroxisome (0.42) Other (0.96)	Targeted (10.348)	Peroxisomal	SignalPeptide=No Loc=Other (0.9979)	PredHelix=0	SigPep=No TM-reg=No	**Most likely peroxisomal**
A. polyphaga catalase	LQHA01015299.1	>LQHA01015299.1 [illegible]	protein_length=209	Cytoplasmic (1.530) Mitochondrial (1.128)	Peroxisome (0.7158) soluble (0.9215)	Peroxisome (0.00) Other (0.97)	Not targeted (-36.142)	Mitochondrial	Loc=Other(0.999) SignalPeptide=No	PredHelix=0	SigPep=No TM-reg=No	
A. royreba catalase	CDEZ01019100.1	>CDEZ01019100.1 [illegible]	protein_length=449	Peroxisomal (2.820)	Peroxisome (0.673) soluble (0.9363)	Peroxisome (0.44) Other (0.97)	Targeted (10.348)	Peroxisomal	SignalPeptide=No Loc=Other (0.9984)	PredHelix=0	SigPep=No TM-reg=No	**Most likely peroxisomal**
A. royreba catalase	CDEZ01019971.1	>CDEZ01019971.1 [illegible]	protein_length=275	Peroxisomal (1.109) Cytoplasmic (1.694) Mitochondrial (1.513)	Cytoplasm (0.5611) soluble (0.932)	Peroxisome (0.00) Other (0.97)	Not targeted (-56.370)	Peroxisomal	SignalPeptide=No Loc=Other (0.9945)	PredHelix=0	SigPep=No TM-reg=No	
A. royreba catalase	CDEZ01020527.1	>CDEZ01020527.1 [illegible]	protein_length=741	Peroxisomal (2.060) Cytoplasmic (1.382)	Cytoplasm (0.6963) soluble (0.9948)	Peroxisome (0.00) Other (0.97)	Not targeted (-69.835)	Peroxisomal	SignalPeptide=No Loc=Other (0.9937)	PredHelix=0	SigPep=No TM-reg=No	
A. royreba catalase	CDEZ01023365.1	>CDEZ01023365.1 [illegible]	protein_length=555	Peroxisomal (1.607) Cytoplasmic (1.067)	Peroxisome (0.5701) soluble (0.9455)	Peroxisome (0.00) Other (0.96)	Not targeted (-56.192)	Mitochondrial	SignalPeptide=No Loc=Other (0.9966)	PredHelix=0	SigPep=No TM-reg=No	
B. mandrillaris catalase	LFUI01000100.1	>LFUI01000100.1 [illegible]	protein_length=492	Peroxisomal (2.393)	Peroxisome (0.7234) soluble (0.9493)	Peroxisome (0.99) Other (0.96)	Targeted (6.233)	Peroxisomal	SignalPeptide=No Loc=Other (0.9972)	PredHelix=0	SigPep=No TM-reg=No	**Most likely peroxisomal**
B. mandrillaris catalase	LFUI01000496.1	>LFUI01000496.1 [illegible]	protein_length=491	Peroxisomal (2.360)	Peroxisome (0.7147) soluble (0.9495)	Peroxisome (0.99) Other (0.97)	Targeted (6.233)	Peroxisomal	SignalPeptide=No Loc=Other (0.9968)	PredHelix=0	SigPep=No TM-reg=No	**Most likely peroxisomal**
B. mandrillaris catalase	LEOU01000132.1	>LEOU01000132.1 [illegible]	protein_length=492	Peroxisomal (2.408)	Peroxisome (0.7227) soluble (0.9474)	Peroxisome (0.99) Other (0.96)	Targeted (6.233)	Peroxisomal	SignalPeptide=No Loc=Other (0.9972)	PredHelix=0	SigPep=No TM-reg=No	**Most likely peroxisomal**
B. mandrillaris catalase	LEOU01000020.1	>LEOU01000020.1 [illegible]	protein_length=500	Cytoplasmic (1.998) Mitochondrial (1.004)	Peroxisome (0.5635) soluble (0.9363)	Peroxisome (0.00) Other (0.96)	Not targeted (-80.254)	Cytoplasmic	SignalPeptide=No Loc=Other (0.9981)	PredHelix=0	SigPep=No TM-reg=No	
N. fowleri catalase	NF0104910	>NF0104910 [illegible]	protein_length=572	Cytoplasmic (2.362)	Peroxisome (0.4601) soluble (0.8746)	Peroxisome (0.00) Other (0.96)	Not targeted (-30.352)	Cytoplasmic	SignalPeptide=No Loc=Other (0.9988)	PredHelix=0	SigPep=No TM-reg=No	
N. fowleri catalase	NF0121200	>NF0121200 [illegible]	protein_length=488	Peroxisomal (2.626)	Peroxisome (0.726) soluble (0.9429)	Peroxisome (0.00) MTP (0.77) Other (0.23)	Twilight zone (-7.697)	Mitochondrial	SignalPeptide=No Loc=Other (0.999)	PredHelix=0	SigPep=No TM-reg=No	**Likely peroxisomal**
N. gruberi catalase	D2VAE1	>D2VAE1 [illegible]	protein_length=618	Cytoplasmic (1.777) Nuclear (1.762)	Nucleus (0.3172) soluble (0.8569)	Peroxisome (0.00) Other (0.96)	Not targeted (-28.276)	Cytoplasmic	SignalPeptide=No Loc=Other (0.9983)	PredHelix=0	SigPep=No TM-reg=No	
N. gruberi catalase	D2VWQ6	>D2VWQ6 [illegible]	protein_length=489	Peroxisomal (2.820)	Peroxisome (0.7321) soluble (0.9443)	Peroxisome (0.00) Other (0.96)	Not targeted (-22.819)	Mitochondrial	SignalPeptide=No Loc=Other (0.9987)	PredHelix=0	SigPep=No TM-reg=No	
N. lovaniensis catalase	PYSW01000023.1	>PYSW01000023.1 [illegible]	protein_length=489	Peroxisomal (2.601)	Peroxisome (0.6441) soluble (0.9282)	Peroxisome (0.00) Other (0.96)	Twilight zone (-7.697)	Peroxisomal	SignalPeptide=No Loc=Other (0.9973)	PredHelix=0	SigPep=No TM-reg=No	**Most likely peroxisomal**
N. lovaniensis catalase	PYSW01000026.1	>PYSW01000026.1 [illegible]	protein_length=614	Cytoplasmic (2.221)	Peroxisome (0.3543) soluble (0.8743)	Peroxisome (0.00) Other (0.97)	Not targeted (-30.352)	Cytoplasmic	SignalPeptide=No Loc=Other (0.9976)	PredHelix=0	SigPep=No TM-reg=No	
