# Supplementary material for: Ultrastructural, Cytochemical, and Comparative Genomic Evidence of Peroxisomes in Three Genera of Pathogenic Free-Living Amoebae, Including the First Morphological Data for the Presence of This Organelle in Heteroloboseans
Source: Genome Biol Evol. 2020 Jun 30;12(10):1734–50. doi: 10.1093/gbe/evaa129 (PMC7549135; doi:10.1093/gbe/evaa129)
Supplement: evaa129_Supplementary_Data [file evaa129_supplementary_data.zip › evaa129-suppl_data/Supplementary text 1.pdf]

**Supplementary Text 1**

Sequences were aligned using the EMBOSS Needle software.

**1. Alignment between the predicted *Acanthamoeba castellanii* Pex11a (ACA1_053960) and Pex11b (ACA1_297780) protein sequences**

```
#=======================================
#
# Aligned_sequences: 2
# 1: PEX11a
# 2: PEX11b
# Matrix: EBLOSUM62
# Gap_penalty: 10.0
# Extend_penalty: 0.5
#
# Length: 292
# Identity:      59/292 (20.2%)
# Similarity:   101/292 (34.6%)
# Gaps:          74/292 (25.3%)
# Score: 159.5
#
#=======================================


PEX11a             1 -----------MSLSKSCKFLAATDGRDKLYKFFHYGARFLSWFCLNTAN     39
                                :...::.||.....|:|:|.:.|.|..|...| .|....
PEX11b             1 MASTGISPFPPIRFDQAVKFANDASGKDRLLRLFIYVGRLAMW-ALKRRG     49

PEX11a            40 NAQWAKYWSNIDSVMSDGRKLLRLFKFLSEIEKLGTIRE-------ARPM     82
                     .|:.|.....:||..:..|::|||.|..:.::.....|.       .:..
PEX11b            50 LAEAAARIKALDSAFNHTRRVLRLGKLGNAVKQWNDCRAKFWGPAGLKWA     99

PEX11a            83 LLVANLLKTLGMAGYFFFNNLSWAMKFNIV-SGDEKKWGKLSFWAWTVGL    131
                     ..|..|.|......|...::|.|..:..:. |.|.||||..|.|.|..||
PEX11b           100 FFVVELFKITAGNVYICCDHLRWLGEIGVFKSIDWKKWGDRSTWCWFAGL    149

PEX11a           132 LFALVLDVVKYRENSRRQQKALASSSQTEL-----------------AQL    164
                     :.:|.:|.::.|.:..|::..|.....::|                 ||.
PEX11b           150 VGSLAMDALQLRASLTRERLLLQDLKDSQLQPHQLDQLDQRLTTSGDAQA    199

PEX11a           165 KKEQ----------------RELEYAMIR----------EVANLQISTSL    188
                     |.:|                :|.|.|:.:          :||.......|
PEX11b           200 KSQQENKGTAAAATATTATKQEAEKALAQLRRARELIYWDVAKNVWDAPL    249

PEX11a           189 VEINPIKSAGVVG----LAGVVEAGLASYQIWKKC-------    219
                     ..:...|..|:.|    :.|.:.:.:..|..|:..
PEX11b           250 AVVGSFKLTGMPGGVLEVCGTMSSLINVYLSWRAMFPPPAPK    291


#---------------------------------------
#---------------------------------------
```

**2. Alignment between the predicted *Acanthamoeba castellanii* Pex11a (ACA1_053960) and Pex11c (ACA1_279800) protein sequences**

```
#=======================================
#
# Aligned_sequences: 2
# 1: PEX11a
# 2: PEX11c
# Matrix: EBLOSUM62
# Gap_penalty: 10.0
# Extend_penalty: 0.5
#
# Length: 1650
# Identity:      58/1650 ( 3.5%)
# Similarity:   105/1650 ( 6.4%)
# Gaps:        1436/1650 (87.0%)
# Score: 172.5
#
#=======================================


PEX11a             1 -------MSLSKSCKFLAATDGRDKLYKFFHYGARFLSWFCLNTANNAQW     43
                            ..::.:.|.|:...||||:.|..||.:|...|...|.....: 
PEX11c             1 MQDPNFLKYINTTIKLLSTHSGRDKVAKTLHYASRIWIWHLQNKGKKER-     49

PEX11a            44 AKYWSNIDSVMSDGRKLLRLFKFLSEIEKLGTI---------REA---RP     81
                     |....:....:.:.|::.|.|..|:.|..:..:         ||:   |.
PEX11c            50 ADQIESFRQAIGNSRRVGRFFSLLNSIPSIYQLVFPDPRTKDRESDLFRF     99

PEX11a            82 MLLVANLLKTLGMAGYFFFNNLSWAMKFNIVSGDEKK---WGKL-SFWAW    127
                     :|:|||:...|    |:..:||::|.|:..:....:.   |.:| ..|.|
PEX11c           100 LLVVANVSDML----YYVSDNLTYAAKYGFIKLSPETNYFWEELVGSWTW    145

PEX11a           128 TVGLLFALVLDVVKY---------RENSRRQQKALASSSQTELAQLKKEQ    168
                     .|.::..:..|:..|         .|:...:....|.:|.:.||...:|.
PEX11c           146 FVSMIVYIAHDIKTYLKLQQQRLRLEHQVMKDSTTAKASASALAATDEEI    195

PEX11a           169 RELEYAMIREVANLQISTSLVEINPIKSAGVVGLAGVVEAGL-ASYQIWK    217
                     .....::||.:|::|::......|...|:..|||.||:.|.. |.:..|:
PEX11c           196 FNNRLSLIRNLADMQLAIYFCFPNSTWSSQWVGLFGVINAVTGADFMFWQ    245

PEX11a           218 KC------------------------------------------------    219
                     ..                                                
PEX11c           246 DGRQRVLNKSVHEVTNSVLGRIIEKKKRGIARSHGIQLKKIKKTATRHSS    295

PEX11a           220 --------------------------------------------------    219
                                                                       
PEX11c           296 LEGLMALKTFVGDFLDKGCYNKLLEAVGEDIEARVERLQSYDEINFVWMV    345

PEX11a           220 --------------------------------------------------    219
                                                                       
PEX11c           346 SFFTGFQRLRNSHREQFTAIFYVLAILLILDLSRTRGLAFGRLERTNWGE    395

PEX11a           220 --------------------------------------------------    219
                                                                       
PEX11c           396 RERRQPSGGAGIRLHKTLSLGEKKQAVALPVVSISLPPFSPASTTPTTDP    445
```

```
PEX11a    220  --------------------------------------------------   219
PEX11c    446  LRRFPPPPTSSATATTTSSTSSSSTNTTATSKVGPPLHANDGKPRQQQPP   495
PEX11a    220  --------------------------------------------------   219
PEX11c    496  QATPGGPLDDHAAGIKAKARKQQPKEAHSVQSKESTAAAAAGRNYIIFPS   545
PEX11a    220  --------------------------------------------------   219
PEX11c    546  LAEMADTEAGKKASLSLSDGAPSVQFRRHFLFPEAGAALFSNLTTQACWV   595
PEX11a    220  --------------------------------------------------   219
PEX11c    596  DDREGSTGRAPSKFAWFGDSRSELSSGGVVIKHEEWTRIPLLSELRDKLE   645
PEX11a    220  --------------------------------------------------   219
PEX11c    646  TEFHTTFNAVHCSYFRNGSECSGGCPDDPQSQKTDAVFVCLGQKRNLLLS   695
PEX11a    220  --------------------------------------------------   219
PEX11c    696  PKARQDCGTSSKLPHVRVVMEANAALFVHRTARSLYDYSVPQSEELVTGG   745
PEX11a    220  --------------------------------------------------   219
PEX11c    746  NIMLCFRRLAATPKNTTPTVAGRDPLRSSQSRTEGSQAGDKTKTPSGKGN   795
PEX11a    220  --------------------------------------------------   219
PEX11c    796  TAARNAPTSAKNVIYIDEDDEVLDSQREASTSVKTDDLQDYVGRIFSSRH   845
PEX11a    220  --------------------------------------------------   219
PEX11c    846  DLYSLGLHMTYTTSICGNMEKGADSILLSDPAAPPVNERLLYTCVNNSHE   895
PEX11a    220  --------------------------------------------------   219
PEX11c    896  NQIERAVLQCTDHHYTNKQINVLNDIPVRLVRGWRADYETKSSRSSDKDD   945
PEX11a    220  --------------------------------------------------   219
PEX11c    946  ERMWYRYDGLYSVRHYVDEPSRDDGPPSGRGEGPVFCEFLLTRLPLYPTP   995
PEX11a    220  --------------------------------------------------   219
PEX11c    996  PLPPMAPKRSEPPKPTLPSISSLTSLPPPSSFRDARKIDLRQGDYLSRDY   1045
PEX11a    220  --------------------------------------------------   219
PEX11c    1046 DDDDGVEIIEVKHASQPPHHLHPTSTSSSSSTSQHGQQPRQLLPPTSSLL   1095
PEX11a    220  --------------------------------------------------   219
PEX11c    1096 NPSASSSPLRRLSGSEPPSRMPTSYSQLMPASSPQRQHPQHATAAASAAV   1145
PEX11a    220  --------------------------------------------------   219
PEX11c    1146 VHTAAGHSPLHENRLQRTLATELSRGFFEARRSTTSASGLHPPPPSQSNS   1195
```

```
PEX11a          220 --------------------------------------------------   219

PEX11c         1196 SNLNAAMLMYNLFGVNPVMPQAPPGLAALYPAVTPYSDPQQQQQLSELFR  1245

PEX11a          220 --------------------------------------------------   219

PEX11c         1246 PATTSIPAQHHLAALAPPPSAAQSPMALQPSLYEVPTAHLLFFMNLIPQK  1295

PEX11a          220 --------------------------------------------------   219

PEX11c         1296 DREIAQIMRQKELDNAKPKRKDESHDKKRKRRSKEEASGTKKKGGSSKSN  1345

PEX11a          220 --------------------------------------------------   219

PEX11c         1346 GRSRRSTVDEEYIDLGLVDNEDDEIINNWSSRRRGAAVQKKSDVEWEKVK  1395

PEX11a          220 --------------------------------------------------   219

PEX11c         1396 ELAAAERSVKRKKSSGGGGSSRHHKAEPVDAEPMRPKEVSPNKRSRSDRK  1445

PEX11a          220 --------------------------------------------------   219

PEX11c         1446 GRGAAEGEDEGSVFCICRSSEEYGFMIACDKCNEWFHGGCVGLTPAEGQE  1495

PEX11a          220 --------------------------------------------------   219

PEX11c         1496 MKTYICPRCHPPNPRKKAFPLATPCPRRSRVGVATKKENENEEDEFVVDE  1545

PEX11a          220 --------------------------------------------------   219

PEX11c         1546 EKIEWNELGEVVYCGDGDMTDDFQSDDPSGFVFVAYGSFDDIAREREEAR  1595

PEX11a          220 --------------------------------------------------   219

PEX11c         1596 KTREDYVKKQQQQRYLQQRLQAIIKQDNAATNGNPAVEPALEEPTASLHP  1645

#---------------------------------------
#---------------------------------------
```

**3. Alignment between the predicted *Acanthamoeba castellanii* Pex11b (ACA1_297780) and Pex11c (ACA1_279800) protein sequences**

```
#=======================================
#
# Aligned_sequences: 2
# 1: PEX11b
# 2: PEX11c
# Matrix: EBLOSUM62
# Gap_penalty: 10.0
# Extend_penalty: 0.5
#
# Length: 1704
# Identity:      66/1704 ( 3.9%)
# Similarity:   120/1704 ( 7.0%)
# Gaps:        1472/1704 (86.4%)
# Score: 128.0
#
#=======================================


PEX11b             1 MASTGISPFPPIRFDQAVKFANDASGKDRLLRLFIYVGRLAMWALKRRGL     50
                     |.......:    .:..:|..:..||:|::.:...|..|:.:|.|:.:|.
PEX11c             1 MQDPNFLKY----INTTIKLLSTHSGRDKVAKTLHYASRIWIWHLQNKGK     46

PEX11b            51 AEAAARIKALDSAFNHTRRVLRLGKLGNAVKQ-----WNDCRAKFWGPAG     95
                     .|.|.:|::...|..::|||.|...|.|::..     :.|.|.|....
PEX11c            47 KERADQIESFRQAIGNSRRVGRFFSLLNSIPSIYQLVFPDPRTKDRES--     94

PEX11b            96 LKWAFFVVELFK--ITAGNV----YICCDHLRWLGEIGVFK-----SIDW    134
                             :||:  :...||    |...|:|.:..:.|..|     :..|
PEX11c            95 --------DLFRFLLVVANVSDMLYYVSDNLTYAAKYGFIKLSPETNYFW    136

PEX11b           135 KKWGDRSTWCWFAGLVGSLAMDALQLRASLTRERLLL--QDLKDSQLQPH    182
                     ::.  ..:|.||..::..:|.| ::....|.::||.|  |.:|||
PEX11c           137 EEL--VGSWTWFVSMIVYIAHD-IKTYLKLQQQRLRLEHQVMKDS-----    178

PEX11b           183 QLDQLDQRLTTSGDAQAKSQQENKGTAAAATATTATKQEA-EKALAQLRR    231
                              ||:              .|:|:|..||.:|. ...|:.:|.
PEX11c           179 ---------TTA--------------KASASALAATDEEIFNNRLSLIRN    205

PEX11b           232 AREL---IYWDVAKNVWDAPLAVVGSFKLTGMPGGVLEVCGTMSSLINVY    278
                     ..::   ||:....:.|        |.:..|:.|.:..|.|.      .:
PEX11c           206 LADMQLAIYFCFPNSTW--------SSQWVGLFGVINAVTGA------DF    241

PEX11b           279 LSWRAMFPPPAPK-------------------------------------    291
                     :.|:........|
PEX11c           242 MFWQDGRQRVLNKSVHEVTNSVLGRIIEKKKRGIARSHGIQLKKIKKTAT    291

PEX11b           292 --------------------------------------------------    291

PEX11c           292 RHSSLEGLMALKTFVGDFLDKGCYNKLLEAVGEDIEARVERLQSYDEINF    341

PEX11b           292 --------------------------------------------------    291

PEX11c           342 VWMVSFFTGFQRLRNSHREQFTAIFYVLAILLILDLSRTRGLAFGRLERT    391
```

```
PEX11b       292 --------------------------------------------------   291

PEX11c       392 NWGERERRQPSGGAGIRLHKTLSLGEKKQAVALPVVSISLPPFSPASTTP   441

PEX11b       292 --------------------------------------------------   291

PEX11c       442 TTDPLRRFPPPPTSSATATTTSSTSSSSTNTTATSKVGPPLHANDGKPRQ   491

PEX11b       292 --------------------------------------------------   291

PEX11c       492 QQPPQATPGGPLDDHAAGIKAKARKQQPKEAHSVQSKESTAAAAAGRNYI   541

PEX11b       292 --------------------------------------------------   291

PEX11c       542 IFPSLAEMADTEAGKKASLSLSDGAPSVQFRRHFLFPEAGAALFSNLTTQ   591

PEX11b       292 --------------------------------------------------   291

PEX11c       592 ACWVDDREGSTGRAPSKFAWFGDSRSELSSGGVVIKHEEWTRIPLLSELR   641

PEX11b       292 --------------------------------------------------   291

PEX11c       642 DKLETEFHTTFNAVHCSYFRNGSECSGGCPDDPQSQKTDAVFVCLGQKRN   691

PEX11b       292 --------------------------------------------------   291

PEX11c       692 LLLSPKARQDCGTSSKLPHVRVVMEANAALFVHRTARSLYDYSVPQSEEL   741

PEX11b       292 --------------------------------------------------   291

PEX11c       742 VTGGNIMLCFRRLAATPKNTTPTVAGRDPLRSSQSRTEGSQAGDKTKTPS   791

PEX11b       292 --------------------------------------------------   291

PEX11c       792 GKGNTAARNAPTSAKNVIYIDEDDEVLDSQREASTSVKTDDLQDYVGRIF   841

PEX11b       292 --------------------------------------------------   291

PEX11c       842 SSRHDLYSLGLHMTYTTSICGNMEKGADSILLSDPAAPPVNERLLYTCVN   891

PEX11b       292 --------------------------------------------------   291

PEX11c       892 NSHENQIERAVLQCTDHHYTNKQINVLNDIPVRLVRGWRADYETKSSRSS   941

PEX11b       292 --------------------------------------------------   291

PEX11c       942 DKDDERMWYRYDGLYSVRHYVDEPSRDDGPPSGRGEGPVFCEFLLTRLPL   991

PEX11b       292 --------------------------------------------------   291

PEX11c       992 YPTPPLPPMAPKRSEPPKPTLPSISSLTSLPPPSSFRDARKIDLRQGDYL  1041

PEX11b       292 --------------------------------------------------   291

PEX11c      1042 SRDYDDDDGVEIIEVKHASQPPHHLHPTSTSSSSSTSQHGQQPRQLLPPT  1091

PEX11b       292 --------------------------------------------------   291

PEX11c      1092 SSLLNPSASSSPLRRLSGSEPPSRMPTSYSQLMPASSPQRQHPQHATAAA  1141
```

```
PEX11b            292 --------------------------------------------------    291

PEX11c           1142 SAAVVHTAAGHSPLHENRLQRTLATELSRGFFEARRSTTSASGLHPPPPS   1191

PEX11b            292 --------------------------------------------------    291

PEX11c           1192 QSNSSNLNAAMLMYNLFGVNPVMPQAPPGLAALYPAVTPYSDPQQQQQLS   1241

PEX11b            292 --------------------------------------------------    291

PEX11c           1242 ELFRPATTSIPAQHHLAALAPPPSAAQSPMALQPSLYEVPTAHLLFFMNL   1291

PEX11b            292 --------------------------------------------------    291

PEX11c           1292 IPQKDREIAQIMRQKELDNAKPKRKDESHDKKRKRRSKEEASGTKKKGGS   1341

PEX11b            292 --------------------------------------------------    291

PEX11c           1342 SKSNGRSRRSTVDEEYIDLGLVDNEDDEIINNWSSRRRGAAVQKKSDVEW   1391

PEX11b            292 --------------------------------------------------    291

PEX11c           1392 EKVKELAAAERSVKRKKSSGGGGSSRHHKAEPVDAEPMRPKEVSPNKRSR   1441

PEX11b            292 --------------------------------------------------    291

PEX11c           1442 SDRKGRGAAEGEDEGSVFCICRSSEEYGFMIACDKCNEWFHGGCVGLTPA   1491

PEX11b            292 --------------------------------------------------    291

PEX11c           1492 EGQEMKTYICPRCHPPNPRKKAFPLATPCPRRSRVGVATKKENENEEDEF   1541

PEX11b            292 --------------------------------------------------    291

PEX11c           1542 VVDEEKIEWNELGEVVYCGDGDMTDDFQSDDPSGFVFVAYGSFDDIARER   1591

PEX11b            292 --------------------------------------------------    291

PEX11c           1592 EEARKTREDYVKKQQQQRYLQQRLQAIIKQDNAATNGNPAVEPALEEPTA   1641

PEX11b            292 ----    291

PEX11c           1642 SLHP   1645


#---------------------------------------
#---------------------------------------
```

## 4. Alignment between the predicted *Acanthamoeba polyphaga* Pex11a (CDFK01220718.1) and Pex11b (LQHA01002590.1) protein sequences

```
#=======================================
#
# Aligned_sequences: 2
# 1: PEX11a
# 2: PEX11b
# Matrix: EBLOSUM62
# Gap_penalty: 10.0
# Extend_penalty: 0.5
#
# Length: 300
# Identity:      59/300 (19.7%)
# Similarity:   101/300 (33.7%)
# Gaps:          82/300 (27.3%)
# Score: 154.5
#
#=======================================


PEX11a             1 -----------MSLSKSCKFLAATDGRDKLYKxFHYGARFLSWFCLNTAN     39
                                :...::.||.....|:|:|.: |.|..|...| .|....
PEX11b             1 MASTGISPFPPIRFDQAVKFANDASGKDRLLRLFIYVGRLAMW-ALKRRG     49

PEX11a            40 NAQWAKYWSNIDSVMSDGRKLLRLFKFLSEIEKLGTIRE-------ARPM     82
                     .|:.|.....:||..:..|::|||.|..:.::.....|.       .:..
PEX11b            50 LAEAAARIKALDSAFNHTRRVLRLGKLGNAVKQWNDCRAKFWGPAGLKWA     99

PEX11a            83 LLVANLLKTLGMAGYFFFNNLSWAMKFNIV-SGDEKKWGKLSFWAWTVGL    131
                     ..|..|.|......|...::|.|..:..:. |.|.||||..|.|.|..||
PEX11b           100 FFVVELFKITAGNVYICCDHLRWLGEIGVFKSIDWKKWGDRSTWCWFAGL    149

PEX11a           132 LFALVLDVVKYRENSRRQQKALASSSQTEL--------------------    161
                     :.:|.:|.::.|.:..|::..|.....::|
PEX11b           150 VGSLAMDALQLRASLARERLLLQDLKDSQLQPHQLDQLDQRLTTTTTTTS    199

PEX11a           162 --AQLKKEQ-------------------RELEYAMIR----------EVA    180
                       ||.|.:|                   :|.|.|:.:          :||
PEX11b           200 GDAQAKSQQADKATAAAAAVTTTTTATKQEAEKALAQLLRARELIYWDVA    249

PEX11a           181 NLQISTSLVEINPIKSAGVVG----LAGVVEAGLASYQIWKKC-------    219
                     .......|..:...|..|:.|    :.|.:.:.:..|..|:..
PEX11b           250 KNVWDAPLAVVGSFKLTGMPGGVLEVCGTMSSLINVYLSWRAMFPPPTPK    299


#---------------------------------------
#---------------------------------------
```

### 5. Alignment between the predicted *Acanthamoeba polyphaga* Pex11a (CDFK01220718.1) and Pex11c (CDFK01199519.1) protein sequences

```
#=======================================
#
# Aligned_sequences: 2
# 1: PEX11a
# 2: PEX11c
# Matrix: EBLOSUM62
# Gap_penalty: 10.0
# Extend_penalty: 0.5
#
# Length: 1188
# Identity:      57/1188 ( 4.8%)
# Similarity:   106/1188 ( 8.9%)
# Gaps:         974/1188 (82.0%)
# Score: 147.0
#
#=======================================


PEX11a             1 -------MSLSKSCKFLAATDGRDKLYKxFHYGARFLSWFCLNTANNAQW     43
                            ..::.:.|.|:...||||:.| .||.:|...|...|.....:
PEX11c             1 MQDPNFLKYINTTIKLLSTHSGRDKVAKTLHYASRIWIWHLQNKGKKER-     49

PEX11a            44 AKYWSNIDSVMSDGRKLLRLFKFLSEIEKLGTI---------REA---RP     81
                     |....:....:.:.|::.|.|..|:.|..:..:         ||:   |.
PEX11c            50 ADQIESFRQAIGNSRRVGRFFSLLNSIPSIYQLVFPDPRTKDRESDLFRF     99

PEX11a            82 MLLVANLLKTLGMAGYFFFNNLSWAMKFNIVSGDEKK---W---------    119
                     :|:|||:...|    |:..:||::|.|:..:....:.   |
PEX11c           100 LLVVANVSDML----YYVSDNLTYAAKYGFIKLSPETNYFWYLLCSRLFC    145

PEX11a           120 ----GKLSF-------------WAWTVGLLFALVLDVVKY---------R    143
                         |..::             |.|.|.::..:..|:..|         .
PEX11c           146 FVFTGAYAYYLDLRCREELVGSWTWFVSMIVYIAHDIKTYLKLQQRLRL    195

PEX11a           144 ENSRRQQKALASSSQTELAQLKKEQRELEYAMIREVANLQISTSLVEINP    193
                     |:...:....|.:|.:.||...:.......::||.:|::|::......|.
PEX11c           196 EHQVMKDSTTAKASASALAATDEDIFNNRLSLIRNLADMQLAIYFCFPNS    245

PEX11a           194 IKSAGVVGLAGVVEA--GLASYQIWKKC----------------------    219
                     ..|:..|||.||:.|  |.|...::...
PEX11c           246 TWSSQWVGLFGVINAVTGAARRFLFFSTELAVFLSSSLGCWLVKSRLEDS    295

PEX11a           220 --------------------------------------------------    219

PEX11c           296 TPLHTSASGWVMQSAASTSTMIKDDPPAPSSASSTTASWGQTPPVDGKIR    345

PEX11a           220 --------------------------------------------------    219

PEX11c           346 TSGGSDFWATARQLQATPAPHLTTSTFFHYYYLNFRLRDTFSGTAIFYVF    395

PEX11a           220 --------------------------------------------------    219

PEX11c           396 AILLILDLSRTRGLAFGRLKRPSRDERERRQPSGGAGVRLHKTLSLGEKK    445

PEX11a           220 --------------------------------------------------    219

PEX11c           446 QAVALPVVSISLPPLSPASTAPTTDLLRRFPPPPTSSSTATTTSSSSSST    495
```

```
PEX11a          220 --------------------------------------------------    219

PEX11c          496 NTTSSKVVDPPLANDGKPRQPPQTAAGGPLDDHAAGVTAKAGRKQQPPKD    545

PEX11a          220 --------------------------------------------------    219

PEX11c          546 SKESTSAAAAAAGRNYIIFPSLAEMADTEAGKKASLSLSDGPPSVQFRRH    595

PEX11a          220 --------------------------------------------------    219

PEX11c          596 FLFPEAGAALFSNLTTQACWVDDREGSTGRAPSKFAWFGDARSELTSGGV    645

PEX11a          220 --------------------------------------------------    219

PEX11c          646 AIKHEEWTRIPLLSELRDKLEIEFHTTFNAVHCSYFRNGSECSGGCPDDP    695

PEX11a          220 --------------------------------------------------    219

PEX11c          696 QSQKTDAVFVCLGQKRNLLLSPKARQDCGTNSKLPHVRVVMEANAALFVH    745

PEX11a          220 --------------------------------------------------    219

PEX11c          746 HTARSLYEYSVPQSEELVTGGNIMLCFRRLAATPKKTTPTVAGRDPLRSS    795

PEX11a          220 --------------------------------------------------    219

PEX11c          796 QSRTEGSQAGDKAKTPSGKGNTAARNAPTSAKNIIYIDEDDEVLDSQRGT    845

PEX11a          220 --------------------------------------------------    219

PEX11c          846 ALWPRTALDAATTHGLSSYPLICPPEASTSVKTDDLQDYVGRIFSSRHDL    895

PEX11a          220 --------------------------------------------------    219

PEX11c          896 YRLGLHMTYTTSICGNMENGADSILLSDPAAPPVNERLLYTCINNSHENQ    945

PEX11a          220 --------------------------------------------------    219

PEX11c          946 IERAVLQFTDHRYAAKQINVLNDVPVRLVRGWRADYETKSSRSSDKDDER    995

PEX11a          220 --------------------------------------------------    219

PEX11c          996 MWYRYDGLYSVRHYVDEPSRDDGQTSGRGEGPVFCEFLLTRLPLYPTPPL   1045

PEX11a          220 --------------------------------------------------    219

PEX11c         1046 PLMAPKRSEPPKPTLPSISSLTSLPPPSSFRDARKIDLRQGDYLSRDYDD   1095

PEX11a          220 --------------------------------------------------    219

PEX11c         1096 DDGVEIIEVKHASQPPHHLHPTSTSSSSSTSQHGQQPRQLLPPTSSLLNP   1145

PEX11a          220 --------------------------------------    219

PEX11c         1146 SVSSSPLRRLSGSEPPSHTPTSYSQLMPASSPQRQHPQ   1183

#--------------------------------------
#--------------------------------------
```

**6. Alignment between the predicted *Acanthamoeba polyphaga* Pex11b (LQHA01002590.1) and Pex11c (CDFK01199519.1) protein sequences**

```
#=======================================
#
# Aligned_sequences: 2
# 1: PEX11b
# 2: PEX11c
# Matrix: EBLOSUM62
# Gap_penalty: 10.0
# Extend_penalty: 0.5
#
# Length: 1244
# Identity:      73/1244 ( 5.9%)
# Similarity:   125/1244 (10.0%)
# Gaps:        1006/1244 (80.9%)
# Score: 122.0
#
#=======================================


PEX11b             1 MASTGISPFPPIRFDQAVKFANDASGKDRLLRLFIYVGRLAMWALKRRGL     50
                     |.......:    .:..:|..:..||:|::.:...|..|:.:|.|:.:|.
PEX11c             1 MQDPNFLKY----INTTIKLLSTHSGRDKVAKTLHYASRIWIWHLQNKGK     46

PEX11b            51 AEAAARIKALDSAFNHTRRVLRLGKLGNAVKQ-----WNDCRAKFWGPAG     95
                     .|.|.:|::...|..::|||.|...|.|::..     :.|.|.|....
PEX11c            47 KERADQIESFRQAIGNSRRVGRFFSLLNSIPSIYQLVFPDPRTKDRES--     94

PEX11b            96 LKWAFFVVELFK--ITAGNV----YICCDHLRWLGEIGVFK-----SIDW    134
                             :||:  :...||    |...|:|.:..:.|..|     :..|
PEX11c            95 --------DLFRFLLVVANVSDMLYYVSDNLTYAAKYGFIKLSPETNYFW    136

PEX11b           135 KKWGDR-----------------------STWCWFAGLVGSLAMDALQLR    161
                     .....|                       .:|.||..::..:|.| ::..
PEX11c           137 YLLCSRLFCFVFTGAYAYYLDLRCREELVGSWTWFVSMIVYIAHD-IKTY    185

PEX11b           162 ASLARERLLL--QDLKDSQLQPHQLDQLDQRLTTTTTTTSGDAQAKSQQA    209
                     ..|.::||.|  |.:|||                   ||:
PEX11c           186 LKLQQQRLRLEHQVMKDS-------------------TTA----------    206

PEX11b           210 DKATAAAAAVT------TTTTATKQEAEKALAQLLRARELIYWDVAKNVW    253
                      ||:|:|.|.|      ...:..:..|:..||        ||:....:.|
PEX11c           207 -KASASALAATDEDIFNNRLSLIRNLADMQLA--------IYFCFPNSTW    247

PEX11b           254 DAPLAVVGSFKLTGMPGGVLEVCGT------MSSLINVYLS-----W--R    290
                             |.:..|:.|.:..|.|.      .|:.:.|:||     |  :
PEX11c           248 --------SSQWVGLFGVINAVTGAARRFLFFSTELAVFLSSSLGCWLVK    289

PEX11b           291 AMFPPPTPK-----------------------------------------    299
                     :.....||.
PEX11c           290 SRLEDSTPLHTSASGWVMQSAASTSTMIKDDPPAPSSASSTTASWGQTPP    339

PEX11b           300 --------------------------------------------------    299

PEX11c           340 VDGKIRTSGGSDFWATARQLQATPAPHLTTSTFFHYYYLNFRLRDTFSGT    389
```

```
PEX11b        300 --------------------------------------------------  299

PEX11c        390 AIFYVFAILLILDLSRTRGLAFGRLKRPSRDERERRQPSGGAGVRLHKTL  439

PEX11b        300 --------------------------------------------------  299

PEX11c        440 SLGEKKQAVALPVVSISLPPLSPASTAPTTDLLRRFPPPPTSSSTATTTS  489

PEX11b        300 --------------------------------------------------  299

PEX11c        490 SSSSSTNTTSSKVVDPPLANDGKPRQPPQTAAGGPLDDHAAGVTAKAGRK  539

PEX11b        300 --------------------------------------------------  299

PEX11c        540 QQPPKDSKESTSAAAAAAGRNYIIFPSLAEMADTEAGKKASLSLSDGPPS  589

PEX11b        300 --------------------------------------------------  299

PEX11c        590 VQFRRHFLFPEAGAALFSNLTTQACWVDDREGSTGRAPSKFAWFGDARSE  639

PEX11b        300 --------------------------------------------------  299

PEX11c        640 LTSGGVAIKHEEWTRIPLLSELRDKLEIEFHTTFNAVHCSYFRNGSECSG  689

PEX11b        300 --------------------------------------------------  299

PEX11c        690 GCPDDPQSQKTDAVFVCLGQKRNLLLSPKARQDCGTNSKLPHVRVVMEAN  739

PEX11b        300 --------------------------------------------------  299

PEX11c        740 AALFVHHTARSLYEYSVPQSEELVTGGNIMLCFRRLAATPKKTTPTVAGR  789

PEX11b        300 --------------------------------------------------  299

PEX11c        790 DPLRSSQSRTEGSQAGDKAKTPSGKGNTAARNAPTSAKNIIYIDEDDEVL  839

PEX11b        300 --------------------------------------------------  299

PEX11c        840 DSQRGTALWPRTALDAATTHGLSSYPLICPPEASTSVKTDDLQDYVGRIF  889

PEX11b        300 --------------------------------------------------  299

PEX11c        890 SSRHDLYRLGLHMTYTTSICGNMENGADSILLSDPAAPPVNERLLYTCIN  939

PEX11b        300 --------------------------------------------------  299

PEX11c        940 NSHENQIERAVLQFTDHRYAAKQINVLNDVPVRLVRGWRADYETKSSRSS  989

PEX11b        300 --------------------------------------------------  299

PEX11c        990 DKDDERMWYRYDGLYSVRHYVDEPSRDDGQTSGRGEGPVFCEFLLTRLPL 1039

PEX11b        300 --------------------------------------------------  299

PEX11c       1040 YPTPPLPLMAPKRSEPPKPTLPSISSLTSLPPPSSFRDARKIDLRQGDYL 1089

PEX11b        300 --------------------------------------------------  299

PEX11c       1090 SRDYDDDDGVEIIEVKHASQPPHHLHPTSTSSSSSTSQHGQQPRQLLPPT 1139
```

```
PEX11b          300 --------------------------------------------   299

PEX11c         1140 SSLLNPSVSSSPLRRLSGSEPPSHTPTSYSQLMPASSPQRQHPQ   1183


#---------------------------------------
#---------------------------------------
```

**7. Alignment between the predicted *Acanthamoeba royreba* Pex11a (CDEZ01020955.1) and Pex11b (CDEZ01023718.1) protein sequences**

```
#=======================================
#
# Aligned_sequences: 2
# 1: PEX11a
# 2: PEX11b
# Matrix: EBLOSUM62
# Gap_penalty: 10.0
# Extend_penalty: 0.5
#
# Length: 314
# Identity:      61/314 (19.4%)
# Similarity:   104/314 (33.1%)
# Gaps:         117/314 (37.3%)
# Score: 161.0
#
#=======================================


PEX11a             1 -------------MSLSKSCKFLAATDGRDKLYKFFHYGARFLSWFC---     34
                                  :...::.||:....|:|:|.:.|.|..|...|..
PEX11b             1 STMTAAPPLLRAPVGFDQTVKFVNDASGKDRLLRLFIYVGRLAMWALKRQ     50

PEX11a            35 -LNSLSNAQWAKYWTNIDGVMSDGRKLLRLYK------------------     65
                      ||.::....|     :||..:..|::|||.|
PEX11b            51 GLNEVAARVKA-----LDGCFNLTRRVLRLGKLGNALKQCNDCYAKFWRQ     95

PEX11a            66 ---FLSEIEKLGTIRESRPGLLVANLLKTLGMAGYFFFNNISWAMKFNII    112
                        ||.|:.|:    .|....||.:.::.||..|  .|.:|:|
PEX11b            96 WAFFLVEVAKV----SSGNAYLVCDHMRWLGEIG--VFKSINW-------    132

PEX11a           113 SGDEKKWGRLSFWAWTVGLLFALVLDVVKYRENLRR------------QQ    150
                         |:||..|.|.|.:|||.:|.:|.::.|.:|.|            |.
PEX11b           133 ----KRWGDWSTWCWFIGLLGSLAMDALQLRASLARELLLQHDLRESQQP    178

PEX11a           151 KAITLNSQTDLAQLKKEQRELQFAIIREVAN-----------LQISTSLV    189
                     ..:..:..:|..:.:.|:.|.:....|:.|.           .|:..||.
PEX11b           179 HQLDQHGGSDEDEEEDEEEEEESVKARKRARKEEEKKKREKVRQLEASLK    228

PEX11a           190 EINPIRN---------------------------NGIVGLAGVVEAALAS    212
                     ::...|.                           .|::...|.|.:.:..
PEX11b           229 QLRQARELIYWDVAKNVWDAPLAVVGSFKLEGMPGGVLEFCGTVSSLINV    278

PEX11a           213 YQIWKKC-------    219
                     |..|:..
PEX11b           279 YLSWRAMFPPPPIK    292


#---------------------------------------
#---------------------------------------
```

**8. Alignment between the predicted *Acanthamoeba royreba* Pex11a (CDEZ01020955.1) and Pex11c (CDEZ01017628.1) protein sequences**

```
#=======================================
#
# Aligned_sequences: 2
# 1: PEX11a
# 2: PEX11c
# Matrix: EBLOSUM62
# Gap_penalty: 10.0
# Extend_penalty: 0.5
#
# Length: 258
# Identity:      59/258 (22.9%)
# Similarity:   105/258 (40.7%)
# Gaps:          52/258 (20.2%)
# Score: 174.0
#
#=======================================


PEX11a             1 -------MSLSKSCKFLAATDGRDKLYKFFHYGARFLSWFCLNSLSNAQW     43
                            ..|:.:.|.|:...||||:.|.|||.:|.|.|. |.:....|.
PEX11c             1 MQDPQVMKYLNTTIKLLSTHSGRDKVAKTFHYASRILIWH-LQNRGKKQR     49

PEX11a            44 AKYWTNIDGVMSDGRKLLRLYKFLSEIEKLGTIRESRPG-----------     82
                     |.........:.:.|::.|.:.:::.|..:..:....|.
PEX11c            50 ADQVELFRQAIGNSRRVGRFFSYINSIPSIYQLAFPDPRTKDNESDLFRF     99

PEX11a            83 -LLVANLLKTLGMAGYFFFNNISWAMKFNIISGDEKK---WGRL-SFWAW    127
                      |:|||:....    |:..:|:::|.|:|.|....:.   |..| ..|.|
PEX11c           100 LLIVANISDMF----YYISDNLTYAAKYNFIKLSPETNYFWEELVGSWTW    145

PEX11a           128 TVGLLFALVLDVVKYRENLRRQQKAITLNSQTD-------LAQLKKEQRE    170
                     .|.::..:..|:..|.: ||.|::.:...:|.|       ||...:|...
PEX11c           146 FVSMVVYIAHDIKTYLK-LRNQRRRLEQQAQKDSTASKAALAATDEEIFS    194

PEX11a           171 LQFAIIREVANLQISTSLVEINPIRNNGIVGLAGVVEAA---------LA    211
                     .:.::||.||::|::......|....:..|||.||:.|.         ::
PEX11c           195 NRLSLIRNVADMQLAIYFCFPNSTWPSHWVGLFGVINAVTGGTILAVFIS    244

PEX11a           212 SYQIWKKC    219
                     |
PEX11c           245 S-------    245


#---------------------------------------
#---------------------------------------
```

**9. Alignment between the predicted *Acanthamoeba royreba* Pex11b (CDEZ01023718.1) and Pex11c (CDEZ01017628.1) protein sequences**

```
#=======================================
#
# Aligned_sequences: 2
# 1: PEX11b
# 2: PEX11c
# Matrix: EBLOSUM62
# Gap_penalty: 10.0
# Extend_penalty: 0.5
#
# Length: 316
# Identity:      63/316 (19.9%)
# Similarity:   117/316 (37.0%)
# Gaps:          95/316 (30.1%)
# Score: 125.0
#
#=======================================


PEX11b                1 STMTAAPPLLRAPVGFDQTVKFVNDASGKDRLLRLFIYVGRLAMWALKRQ     50
                           ...|.:::.   .:.|:|.::..||:|::.:.|.|..|:.:|.|:.:
PEX11c                1 ---MQDPQVMKY---LNTTIKLLSTHSGRDKVAKTFHYASRILIWHLQNR     44

PEX11b               51 GLNEVAARVKALDGCFNLTRRVLRLGKLGNALKQCNDCYAKFWRQWAF--     98
                        |..:.|.:|:........:|||   |:..:.:......|     |.||
PEX11c               45 GKKQRADQVELFRQAIGNSRRV---GRFFSYINSIPSIY-----QLAFPD     86

PEX11b               99 ------------FLVEVAKVSSGNAYLVCDHMRWLGEIGVFK---SINWK    133
                                    ||:.||.:|. ..|.:.|::.:..:....|   ..|:
PEX11c               87 PRTKDNESDLFRFLLIVANISD-MFYYISDNLTYAAKYNFIKLSPETNY-    134

PEX11b              134 RWGDW-STWCWFIGLLGSLAMDA---LQLRASLARELLLQHDLRESQQPH    179
                        .|.:. .:|.||:.::..:|.|.   |:||                .|..
PEX11c              135 FWEELVGSWTWFVSMVVYIAHDIKTYLKLR----------------NQRR    168

PEX11b              180 QLDQHGGSDEDEEEDEEEEEESVKARKRARKEEEKKKREKVRQLEASLKQ    229
                        :|:|..          :::..:.||...|..||....|         |..
PEX11c              169 RLEQQA----------QKDSTASKAALAATDEEIFSNR---------LSL    199

PEX11b              230 LRQAREL---IYWDVAKNVWDAPLAVVGSFKLEGMPGGVLEFCGTVSSLI    276
                        :|...::   ||:....:.|  |...||.|       ||:. ..|..:::
PEX11c              200 IRNVADMQLAIYFCFPNSTW--PSHWVGLF-------GVIN-AVTGGTIL    239

PEX11b              277 NVYLSWRAMFPPPPIK    292
                        .|::|.
PEX11c              240 AVFISS----------    245


#---------------------------------------
#---------------------------------------
```

**10. Alignment between the predicted *Balamuthia mandrillaris* Pex11a (LFUI01000076.1) and Pex11b (LFUI01000221.1) protein sequences**

```
#=======================================
#
# Aligned_sequences: 2
# 1: PEX11a
# 2: PEX11b
# Matrix: EBLOSUM62
# Gap_penalty: 10.0
# Extend_penalty: 0.5
#
# Length: 274
# Identity:      40/274 (14.6%)
# Similarity:    75/274 (27.4%)
# Gaps:         120/274 (43.8%)
# Score: 95.0
#
#=======================================

PEX11a              1 --------------------------------------------------      0

PEX11b              1 LTAAGFQLEDLVKFVGDASAKDRLFRLAVNVCRLGAWACQKGRSTQAAAQ     50

PEX11a              1 --------------------------RLLRFFRFLEEIQRLRTLKETK--     22
                                                ||||..:.......||.:...|
PEX11b             51 TEALKQWLVLLPQRLKKLEDSLNNTRRLLRLGKISTMYWTLRYVLPKKYP    100

PEX11a             23 --PLYILGSIGKSFGMSLYFFFNNLSWLCKYGAIKGEAKKYSDWSMWWWF     70
                        .|..|....|:..::.||:.::|.|....|.|..:..|:...:...|.
PEX11b            101 EITLLALVEALKTLCLTSYFYHDHLKWASSSGVILFDGNKHGYRATCSWA    150

PEX11a             71 IGVLCSFLMNVGRYRENLRKQKRM---LSAQELKEYKNEQKQLQLAMIRE    117
                      ..:|||.:||:..|..:|.:::::   |.|..|:|.|.|:::.|.....|
PEX11b            151 FSLLCSLVMNLLNYMVSLERERQLRTTLQAYLLQEEKAEKQRQQQEKEGE    200

PEX11a            118 LANL---QISSALVDLNPMKSKG---LVGLAGVIEALLGAHAIWKRC---    158
                      .:::   :.|:|.:.    |.||   ......::.:..||....::.
PEX11b            201 RSSVGDEEESAAGIS----KEKGKERQTNTTTLLASTAGAKLDTEKALQK    246

PEX11a            159 ------------------------    158

PEX11b            247 LMEARRLIVLDFIKNVADLPSGVV    270


#---------------------------------------
#---------------------------------------
```

**11. Alignment between the predicted *Naegleria fowleri* Pex11a (NF0113850) and Pex11b (NF0009070) protein sequences**

```
#=======================================
#
# Aligned_sequences: 2
# 1: PEX11a
# 2: PEX11b
# Matrix: EBLOSUM62
# Gap_penalty: 10.0
# Extend_penalty: 0.5
#
# Length: 361
# Identity:      69/361 (19.1%)
# Similarity:   130/361 (36.0%)
# Gaps:         116/361 (32.1%)
# Score: 214.0
#
#=======================================


PEX11a             1 MSQDLKASPYKAVINFINQFQTLTNTGLGRDKATKVIQYGCKIIEEILE-     49
                             ....|..|::||..:.:|..|||:.||::|||.|::...:| 
PEX11b             1 --------MDHPVYIFLSQFVKIMDTLSGRDRVTKILQYGAKVVSYAIET     42

PEX11a            50 --------------RSASPTVDHK--DL----------IVRVQRTSAGLA     73
                                   .|:...:.|:  ||          :.|.....:.::
PEX11b            43 RRQLVLKHLISEHVESSHSLIHHEAFDLEKTKTFYTQALKRTSNFESSIS     92

PEX11a            74 TARRVMRFWKPFQG----YVALIQFIE-----------------------     96
                     .||:|.||:|....    |..:|||.:                       
PEX11b            93 DARKVFRFFKSIGSLLDVYKFVIQFYQLHWKKRETNHTSSTTGNATNSTN    142

PEX11a            97 ALISGKKQTVTAILDLV---SKLCMAHYFLVDHLTWLSREKILSDMPLEL    143
                     .:.:|:|:.....:|.|   .|..::.|.|.||.:|.::..:..|: .||
PEX11b           143 EMKNGEKRPTLRWIDFVRVSQKFLLSIYILYDHASWAAKAGLFYDV-TEL    191

PEX11a           144 AQKSQSFFASTFNNNKNADYSRIGSNFWFYGVVFAILAHVLRWSEYLTKE    193
                       .:...:...|||::...||......||.|.:..:::.|..:.:...:|
PEX11b           192 --NAVHVWQKLFNNSRAKHYSETSCKMWFIGTLLMLISDVYDFLDTFNEE    239

PEX11a           194 ----KKEFDIVRDA------------------------------------    203
                         :::.|.:|:.                                    
PEX11b           240 IRCLREKADCMRELPEGFLNLSDFLVEPSNAAAVPTPSNPIKNSKLSSID    289

PEX11a           204 --------NQQKMFRTLIALFCDFGTAAILAKKTTFQNKAALGVFGVVSS    245
                             .:.::.|.:|....|...|.....|....|.|.:|:.|.||:
PEX11b           290 TKLKEISDRKSQIVRNIIKNTSDLLVAGNGGYKVWSLNNAVVGISGCVSA    339

PEX11a           246 LISIYDAWPSQ    256
                     ::.:|:.||.:
PEX11b           340 VVGLYETWPKK    350


#---------------------------------------
#---------------------------------------
```

**12. Alignment between the predicted *Naegleria lovaniensis* Pex11a (PYSW01000002.1) and Pex11b (PYSW01000038.1) protein sequences**

```
#=======================================
#
# Aligned_sequences: 2
# 1: PEX11a
# 2: PEX11b
# Matrix: EBLOSUM62
# Gap_penalty: 10.0
# Extend_penalty: 0.5
#
# Length: 348
# Identity:      70/348 (20.1%)
# Similarity:   130/348 (37.4%)
# Gaps:         105/348 (30.2%)
# Score: 220.5
#
#=======================================

PEX11a             1 MSQDLKTSPYKAVINFINQFQTLTNTGLGRDKATKVLQYGCKIIEEILER     50
                             ....:..|::||..:.:|..|||:.||:||||.|::...:|.
PEX11b             1 --------MDHPIYIFLSQFVKIMDTLSGRDRVTKILQYGAKVVSYGIET     42

PEX11a            51 ---------------SASPAVDHK--DL----------IVRVQRTSAGLA     73
                                    |.:..:.|:  ||          :.|.....:.::
PEX11b            43 RRQLVLKHLFSSSSDSTTSLIHHEAFDLEKTKTFYTQALKRTSNFESSIS     92

PEX11a            74 TARRVMRFWKPFQGYVALIQFI--------------EALIGGKKQTVTAI    109
                     .||:|.||:|.....:.:.:|:              |.|  |:|:.....
PEX11b            93 DARKVFRFFKSIGSLLDVYKFVINLYQLHWKKGTNNEKL--GEKRPNLRW    140

PEX11a           110 LDLV---SKLCMAHYFLMDHLTWLSREKILSDMPLELAQKSQSFFASTFN    156
                     :|.|   .|..::.|.|.||.:|.::..:..|: .||  .:...:...||
PEX11b           141 IDFVRVSQKFLLSIYILYDHASWAAKAGLFYDV-TEL--NAVHVWQKLFN    187

PEX11a           157 NNKNADYSRSSSNFWFYGVIFAILAHVLRWSEYLTKE----KKELDIVRD    202
                     |::...||.:|...||.|.:..:::.|..:.:...:|    :::.|.:||
PEX11b           188 NSRAKHYSETSCKMWFIGTLLMLISDVYDFFDTFNEEIRCLREKADCMRD    237

PEX11a           203 A--------------------------------------------NQQKM    208
                     .                                            .:.::
PEX11b           238 LPEGFLNLSDFLVEPSNAAAVPTPSNPIKNSRLSSIDAKLKEISDRKSQI    287

PEX11a           209 FRTFIALLCDFGTAAILAKKTSFQNKAALGVFGVVSSLISIYDAWPSQ    256
                     .|..|....|...|.....|....|.|.:|:.|.||:::.:|:.||:.
PEX11b           288 VRNIIKNTSDLLVAGNGGYKVWSLNNAVVGISGCVSAVVGLYETWPAP    335


#---------------------------------------
#---------------------------------------
```

### 13. Alignment between the predicted *Naegleria gruberi* Pex11a (D2V0G7) and Pex11b (ACER01000200.1) protein sequences

```
#=======================================
#
# Aligned_sequences: 2
# 1: PEX11a
# 2: PEX11b
# Matrix: EBLOSUM62
# Gap_penalty: 10.0
# Extend_penalty: 0.5
#
# Length: 363
# Identity:      80/363 (22.0%)
# Similarity:   128/363 (35.3%)
# Gaps:         119/363 (32.8%)
# Score: 219.5
#
#=======================================


PEX11a             1 MSTTASSP-YKSAINFINQFQTLTNSGLGRDKATKIIQYGCKII------     43
                         ..:| |...:.|.....||:    ||||.||.:|||.|||      
PEX11b             1 ----MDNPIYIKLLQFNKVMDTLS----GRDKVTKALQYGAKIISYSLDL     42

PEX11a            44 --EEILEKSASP------ANDYKDL----------IVRVQRTSAGLATAR     75
                       :.||....|.      .|:..||          :.||....:.::.||
PEX11b            43 KKQSILSNLNSSNISNLLENELLDLDKQKIFYTRSLKRVSNFESSISDAR     92

PEX11a            76 RVMRFWKPFQGYVALLQFIESL----------------------------     97
                     :|.||.|.....:.:.:||.:|                            
PEX11b            93 KVFRFAKSIGSLLDVYKFIINLYKFYLKKYLFKNNLNNNNNLNELNNLNN    142

PEX11a            98 INGKKQTVIGIFELVSKLCMAHYFLMDHLTWLSKEKILSELPLELAEKSQ    147
                     :|..|...|....::.|..::.|.|.||.:|.:|.::.|::. ||  .:.
PEX11b           143 LNEFKVKWIDFIRILQKFLLSIYILYDHASWAAKVQLFSDVN-EL--NAS    189

PEX11a           148 SFIATTFNNNKNADYSRSSSNFWFYGVVFALVAHVLKY------------    185
                     ......|||::..:||.:|...||.|.:..|::....|            
PEX11b           190 HLYQKLFNNSRAKNYSETSCKMWFIGTLLTLISDFYDYFDIFNQEISILR    239

PEX11a           186 ----------------SEYLTKEKK----------------EFDITR---    200
                                     ::::..:||                ...|||   
PEX11b           240 EKSNSISQLNEGFINLNDFIDHDKKTSAEIAIPSNNNLNNNNSTITRISQ    289

PEX11a           201 ------DVNQQK--MFRTFIALVCDLGTAAILAKKISYQNKAALGVFGVA    242
                           .|..:|  :.|..:....||..|.....|:...|...:|:.|..
PEX11b           290 IDERLKKVQDKKVQITRNLMKNTADLMVAGNGGYKVWSLNNGIIGLCGCI    339

PEX11a           243 SSLISIYDNWPSQ    255
                     ||.|.:|::|||.
PEX11b           340 SSFIGLYESWPSN    352


#---------------------------------------
#---------------------------------------
```
