# Supplementary material for: Ultrastructural, Cytochemical, and Comparative Genomic Evidence of Peroxisomes in Three Genera of Pathogenic Free-Living Amoebae, Including the First Morphological Data for the Presence of This Organelle in Heteroloboseans
Source: Genome Biol Evol. 2020 Jun 30;12(10):1734–50. doi: 10.1093/gbe/evaa129 (PMC7549135; doi:10.1093/gbe/evaa129)
Supplement: evaa129_Supplementary_Data [file evaa129_supplementary_data.zip › evaa129-suppl_data/Supplementary text 2.pdf]

Supplementary Text 2

Alignment of the predicted *Acanthamoeba polyphaga* Pex12 CDFK01222939.1 and CDFK01184659.1 protein sequences. The sequences were aligned using EMBOSS Needle.

```
CDFK01222939       1 MANFSSGEAQRPSFFEMVAQQELLPFFGPALKYALSVAAVRWPRLEWGVT     50

CDFK01184659       1 --------------------------------------------------      0

CDFK01222939      51 HHDELFYGLRLLLEAHHLRRHDASFSEHFYSLKRVRVVAPTTDSGAPSRS    100

CDFK01184659       1 --------------------------------------------------      0

CDFK01222939     101 PATAGSRLTDHDRRVSLLLLVGLPYAKAKLDQLHKRMAGPLSGWLETEGS    150

CDFK01184659       1 --------------------------------------------------      0

CDFK01222939     151 DGNDNDDDNDEGDGGEGEGAAAEDGGRRARWWRRWRRVMRVARGLFVAGY    200

CDFK01184659       1 --------------------------------------------------      0

CDFK01222939     201 PWASAVYEGLFFVYQVLYLYDHTRYYTPFLHLQRLQVQRLSLEDTIEMTQ    250

CDFK01184659       1 --------------------------------------------------      0

CDFK01222939     251 DTARRRAAGAESWGVGDARGAAAAVRTVGRVLARAWHVVEDYSALALPLV    300

CDFK01184659       1 --------------------------------------------------      0

CDFK01222939     301 LFVFKFLEWWYAENTKQAAPALPTPP------------------------    326
                                       ||||||||
CDFK01184659       1 ------------------APALPTPPPPRPPPVVEEAGRKKPDGECGLCG     32

CDFK01222939     327 --------------------------------------------------    326

CDFK01184659      33 KRRTNPAMVAGTGYVFCYPCLHASVTTHGRCPATGLPASPDTLLKLYEST     82


#---------------------------------------
#---------------------------------------
```
