# Supplementary figures and images for: Ultrastructural, Cytochemical, and Comparative Genomic Evidence of Peroxisomes in Three Genera of Pathogenic Free-Living Amoebae, Including the First Morphological Data for the Presence of This Organelle in Heteroloboseans
Source: Genome Biol Evol. 2020 Jun 30;12(10):1734–50. doi: 10.1093/gbe/evaa129 (PMC7549135; doi:10.1093/gbe/evaa129)

# BUSCO Assessment Results

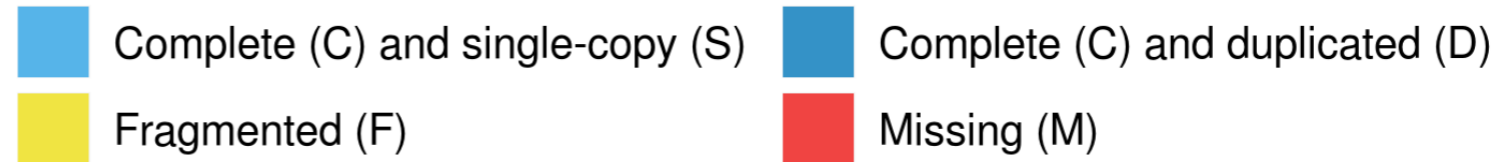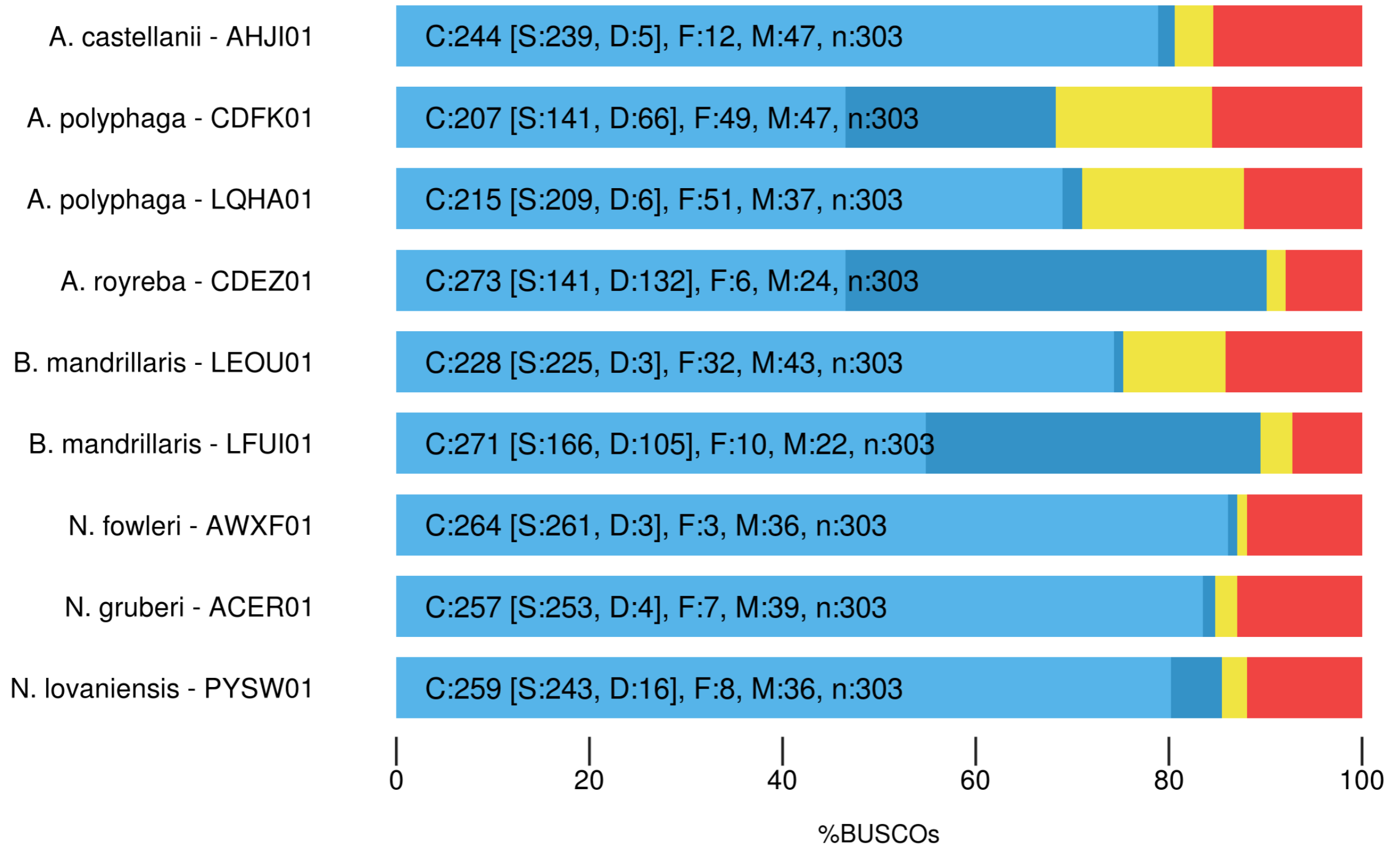

Supplement: evaa129_Supplementary_Data [file evaa129_supplementary_data.zip › evaa129-suppl_data/Supplementary figure 1.pdf]
